# Supplementary material for: Selective production of methylindan and tetralin with xylose or hemicellulose
Source: Nat Commun. 2024 May 2;15:3723. doi: 10.1038/s41467-024-48101-x (PMC11066016; doi:10.1038/s41467-024-48101-x)
Supplement: Supplementary file 1 — Supplementary information [file 41467_2024_48101_MOESM1_ESM.pdf]

Supporting information

Selective production of methylindan and tetralin with xylose or hemicellulose

Zhufan Zou[1,2,3,†], Zhenjie Yu[1,3,†], Weixiang Guan[1], Yanfang Liu,[4] Yumin Yao,[4] Yang Han,[4] Guangyi Li[1], Aiqin Wang[1,5], Yu Cong[1], Xinmiao Liang[4]*, Tao Zhang[1,2,5]* & Ning Li[1]*

[1] CAS Key Laboratory of Science and Technology on Applied Catalysis, Dalian Institute of Chemical Physics, Chinese Academy of Sciences, Dalian 116023, China.

[2] School of Chemistry, Dalian University of Technology, Dalian 116024, China.

[3] University of Chinese Academy of Sciences, Beijing 100049, China.

[4] Key Lab of Separation Science for Analytical Chemistry, Dalian Institute of Chemical Physics, Chinese Academy of Sciences, Dalian 116023, China.

[5] State Key Laboratory of Catalysis, Dalian Institute of Chemical Physics, Chinese Academy of Sciences, Dalian 116023, China.

[†] These authors contributed equally: Zhufan Zou, Zhenjie Yu.

* email: liangxm@dicp.ac.cn; taozhang@dicp.ac.cn; lining@dicp.ac.cn

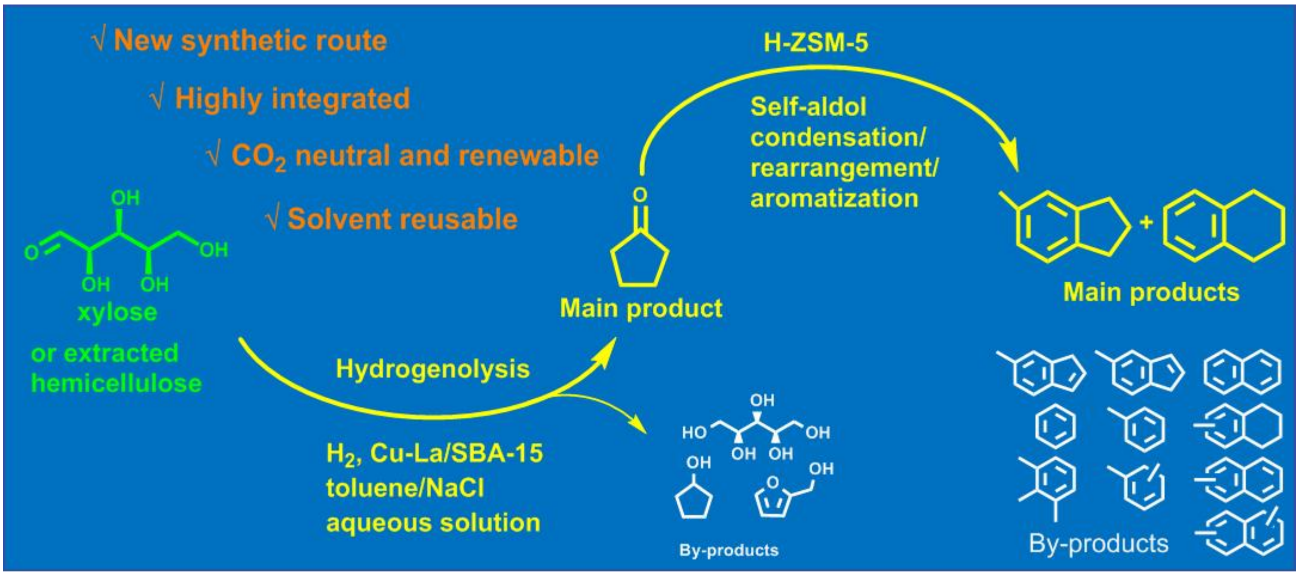

Supplementary Fig. 1 Strategies for the selective production of methylindan and tetralin from xylose or the hemicellulose extracted from raw biomass.

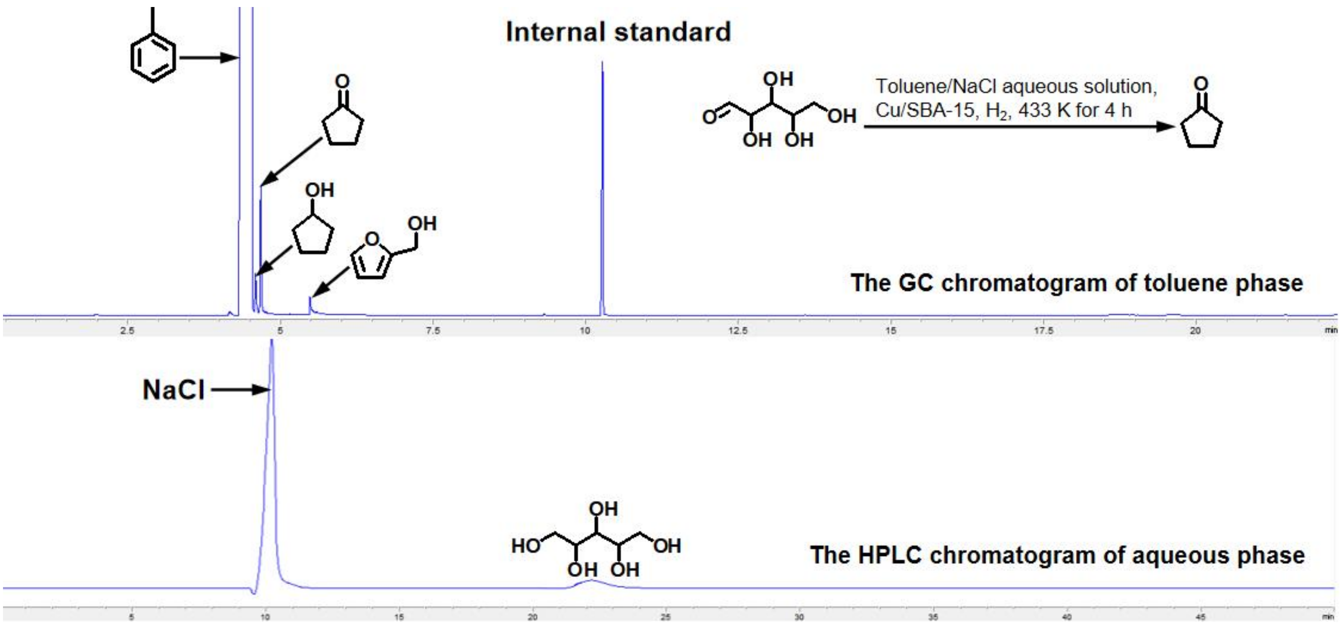

Supplementary Fig. 2 GC and HPLC chromatograms of the products obtained from the hydrogenolysis of xylose over the Cu/SBA-15 catalyst. Reaction conditions: 433 K, 3 MPa H_2, 4 h, 500 rpm; 10 mL toluene, 10 mL 5 wt.% NaCl aqueous solution, 0.3 g xylose and 30 mg catalyst were used for the test.

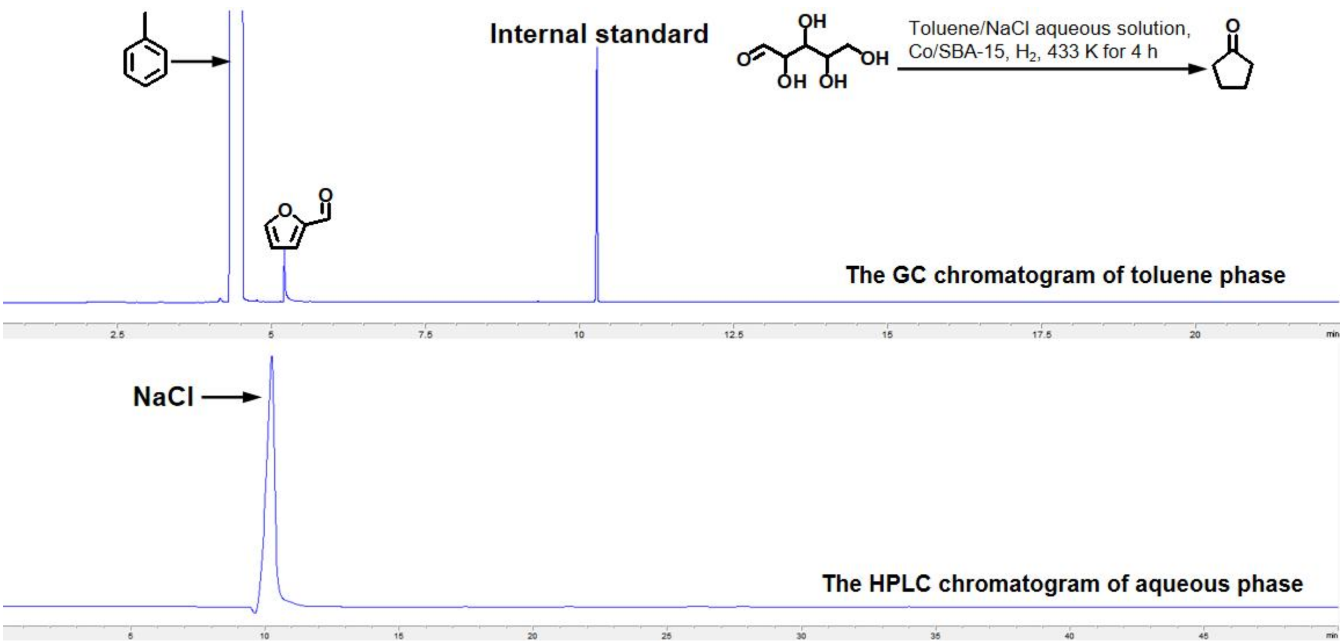

Supplementary Fig. 3 GC and HPLC chromatograms of the products obtained from the hydrogenolysis of xylose over the Co/SBA-15 catalyst. Reaction conditions: 433 K, 3 MPa H_2, 4 h, 500 rpm; 10 mL toluene, 10 mL 5 wt.% NaCl aqueous solution, 0.3 g xylose and 30 mg catalyst were used for the test.

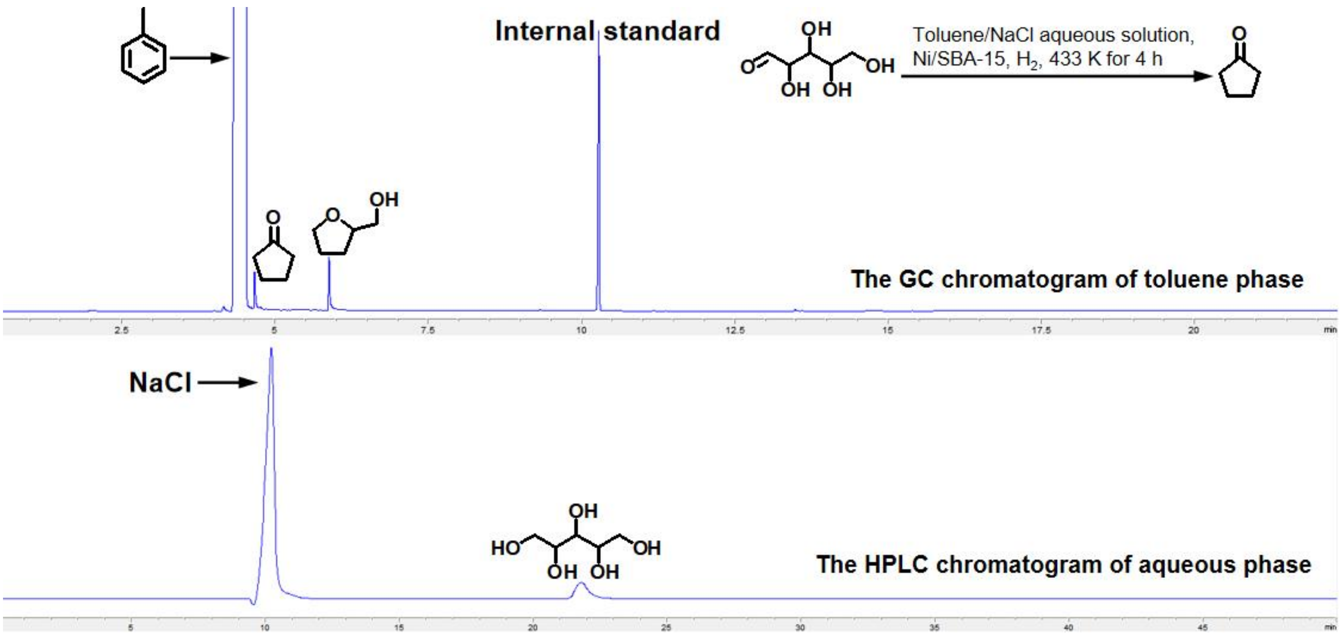

Supplementary Fig. 4 GC and HPLC chromatograms of the products obtained from the hydrogenolysis of xylose over the Ni/SBA-15 catalyst. Reaction conditions: 433 K, 3 MPa H_2, 4 h, 500 rpm; 10 mL toluene, 10 mL 5 wt.% NaCl aqueous solution, 0.3 g xylose and 30 mg catalyst were used for the test.

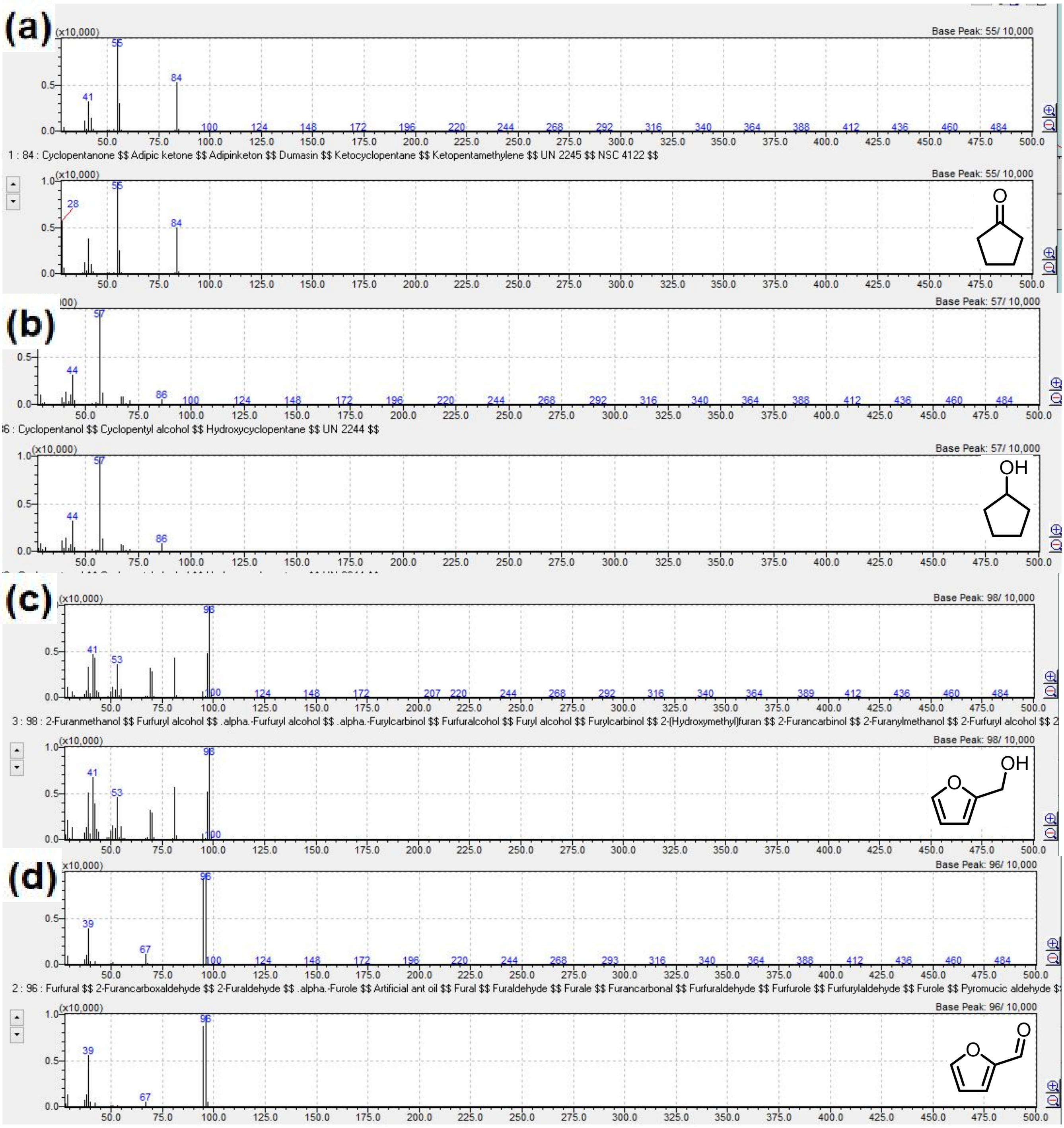

Supplementary Fig. 5 Mass spectrograms of the (a) cyclopentanone (CPO), (b) cyclopentanol (CPL), (c) furfuryl alcohol (FA), and (d) furfural (FF) obtained from the hydrogenolysis of xylose.

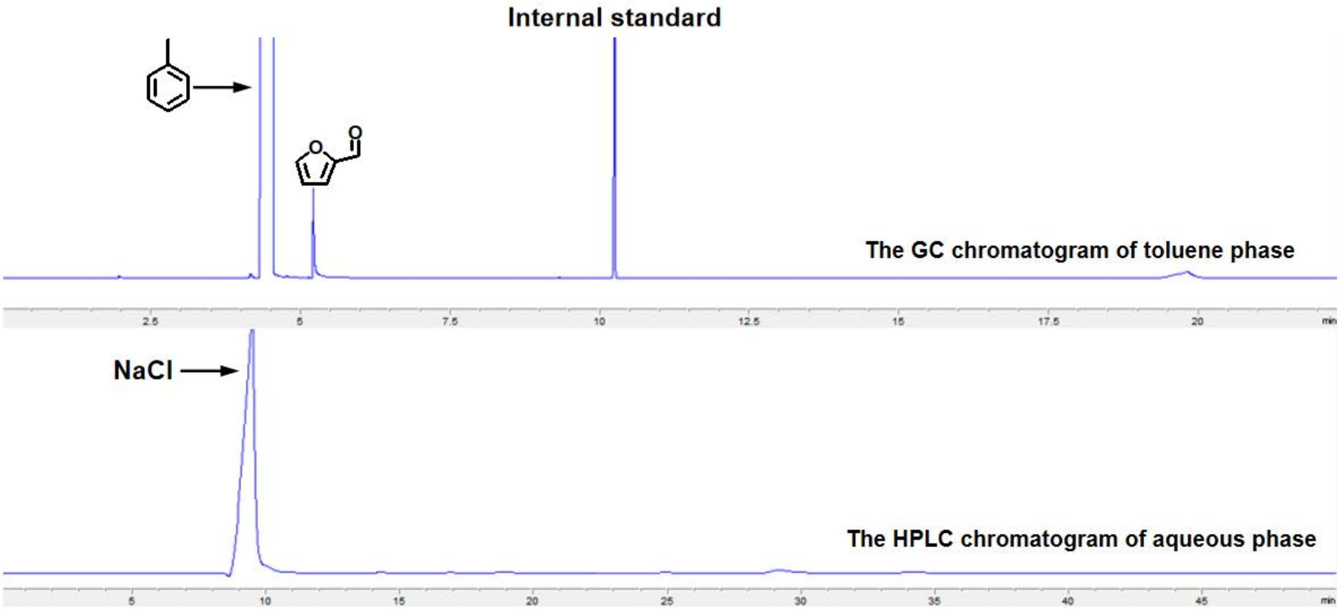

Supplementary Fig. 6 GC and HPLC chromatograms of the products obtained from a hydrogenolysis of xylose in the absence of any catalyst. Reaction conditions: 433 K, 3 MPa H_2, 4 h, 500 rpm; 10 mL toluene, 10 mL 5 wt.% NaCl aqueous solution and 0.3 g xylose were used for the test.

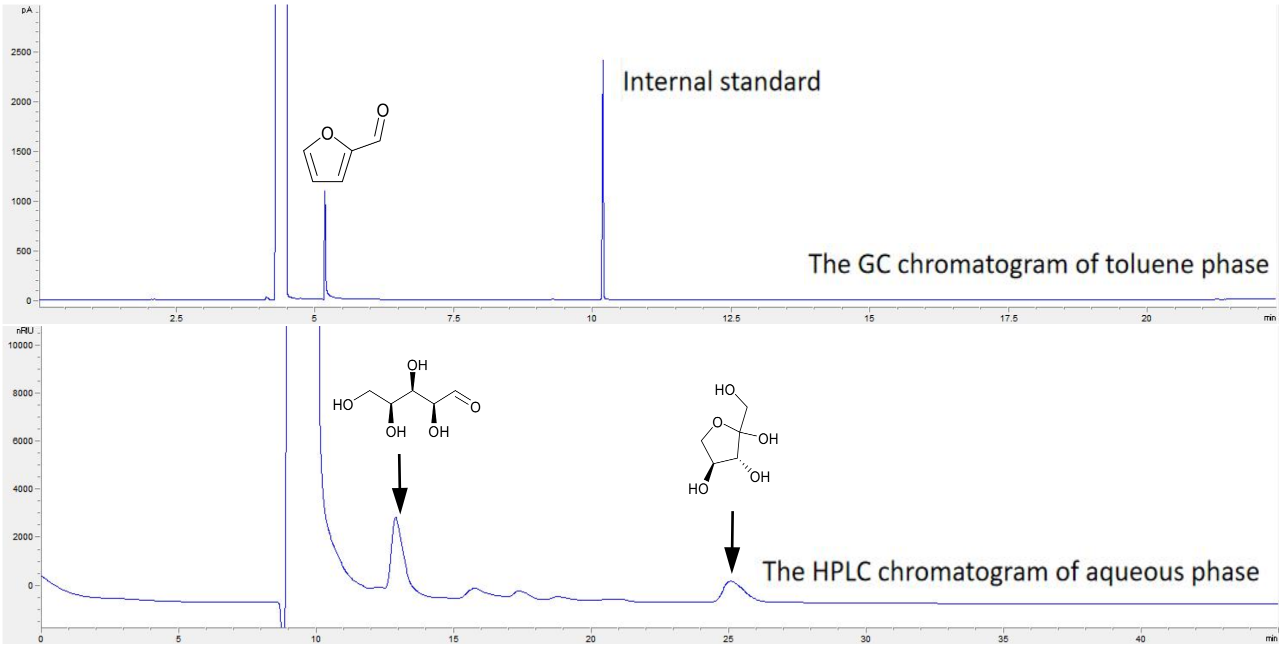

Supplementary Fig. 7 GC and HPLC chromatograms of the products obtained from the reaction of xylose in a toluene/NaCl aqueous solution biphasic reaction system. Reaction conditions: 433 K, 0.5 MPa N_2, 500 rpm, 35 min; 10 mL 5 wt.% NaCl aqueous solutions, 10 mL toluene and 0.3 g xylose were used for the test.

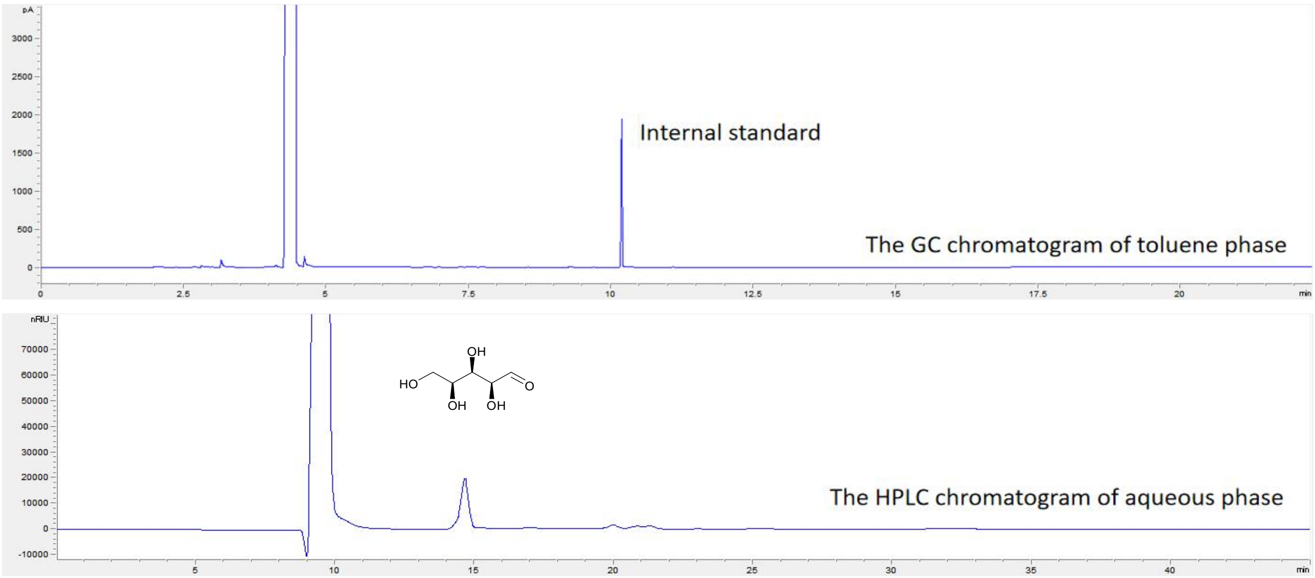

Supplementary Fig. 8 GC and HPLC chromatograms of the products obtained from the reaction of xylose in a toluene/H_2O biphasic reaction system. Reaction conditions: 433 K, 0.5 MPa N_2, 500 rpm, 35 min; 10 mL dionized H_2O, 10 mL toluene and 0.3 g xylose were used for the test.

To figure out the reasons for the significant promotion effect of NaCl, we studied the conversion of xylose in the absence of metal catalyst under the investigated reaction conditions. From the GC and HPLC chromatograms of the products that were obtained in a toluene/NaCl aqueous solution biphasic reaction system at a short reaction time of 35 min (see Supplementary Fig. 7), we can clearly see the peaks of xylulose and furfural. In contrast, we didn't notice these peaks in the GC and HPLC chromatograms of the products that were obtained in a toluene/water biphasic reaction system (see Supplementary Fig. 8). Based on these results, we believe that the presence of NaCl accelerated the isomerization/dehydration of xylose to furfural. This may be one reason for the promotion effect of NaCl.

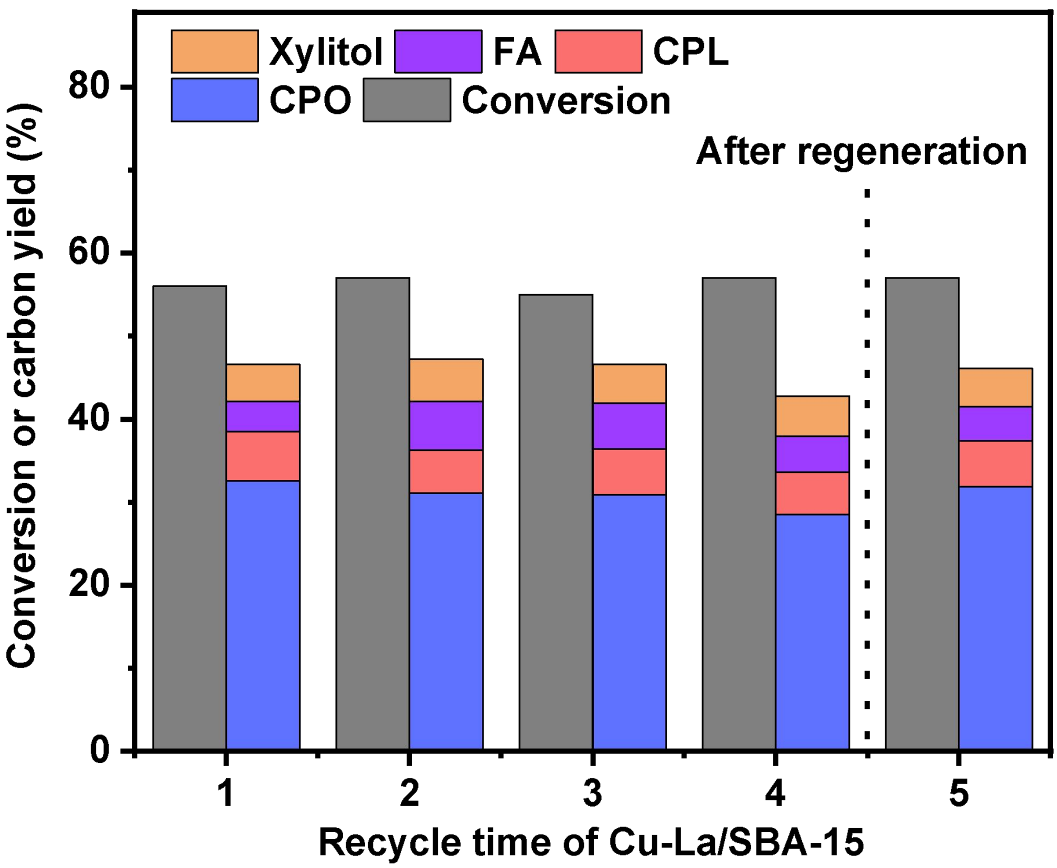

Supplementary Fig. 9 Xylose conversion and the carbon yields of different products over the Cu-La/SBA-15 catalyst as the function of recycle time. Reaction conditions: 438 K, 3 MPa H_2, 500 rpm, 1 h; 10 mL toluene, 10 mL 3 wt.% NaCl aqueous solution, 0.3 g xylose and 30 mg fresh Cu-La/SBA-15 catalyst were used for the test. CPO: cyclopentanone. CPL: cyclopentanol. FA: furfuryl alcohol.

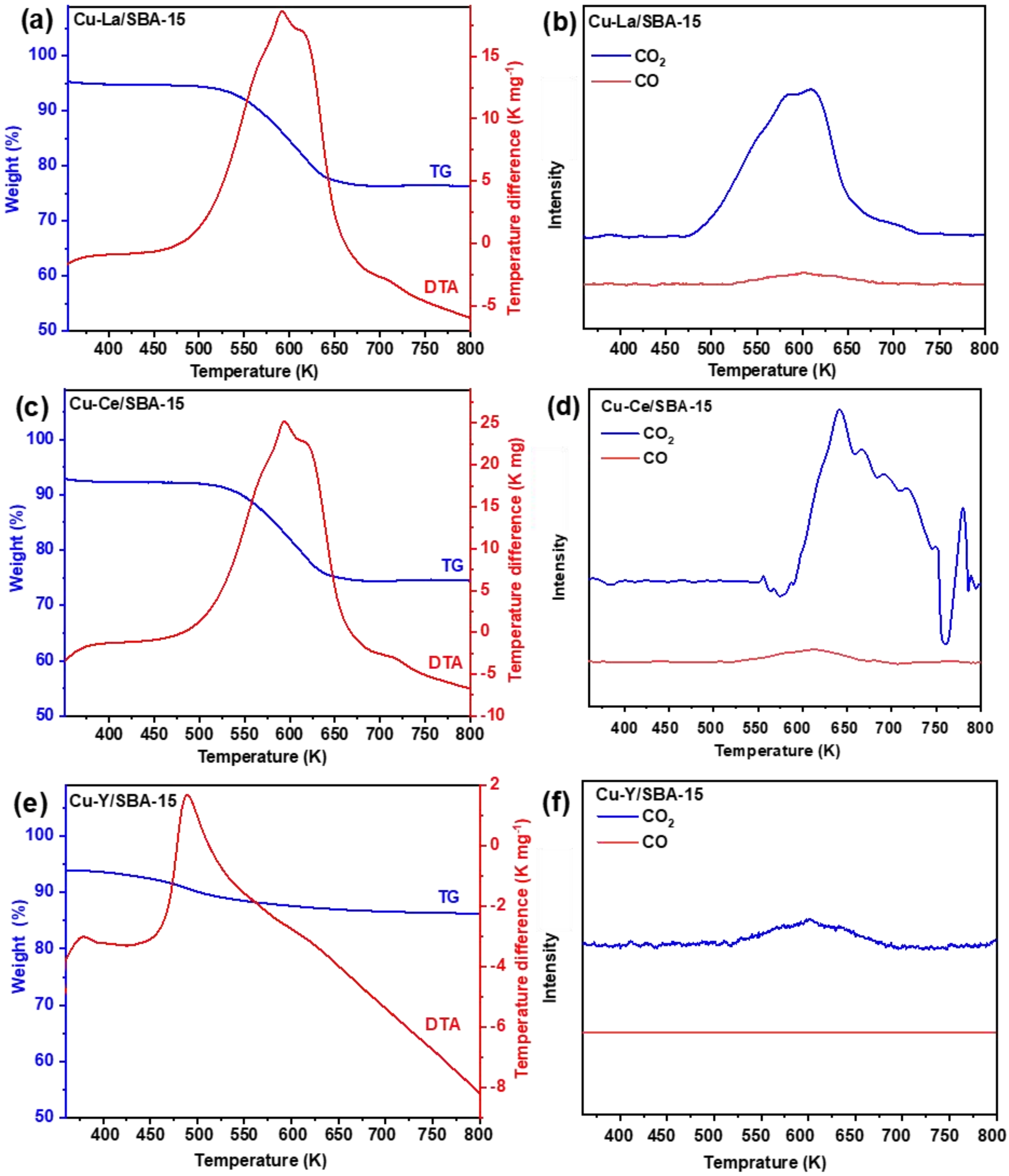

Supplementary Fig. 10 TG-DTA (a, c, e) and synchronous MS (b, d, f) profiles of the used Cu-La/SBA-15, Cu-Ce/SBA-15 and Cu-Y/SBA-15 catalysts.

Supplementary Table 1 Actual metal contents in the fresh and used Cu/SBA-15 or Cu-La/SBA-15 catalysts

Catalyst	Metal content measured by ICP-OES	
	Cu (wt.%)	La (wt.%)
Cu/SBA-15	23.1	—
Used Cu/SBA-15	16.6	—
Cu-La/SBA-15	24.5	3.7
Used Cu-La/SBA-15	22.9	3.7

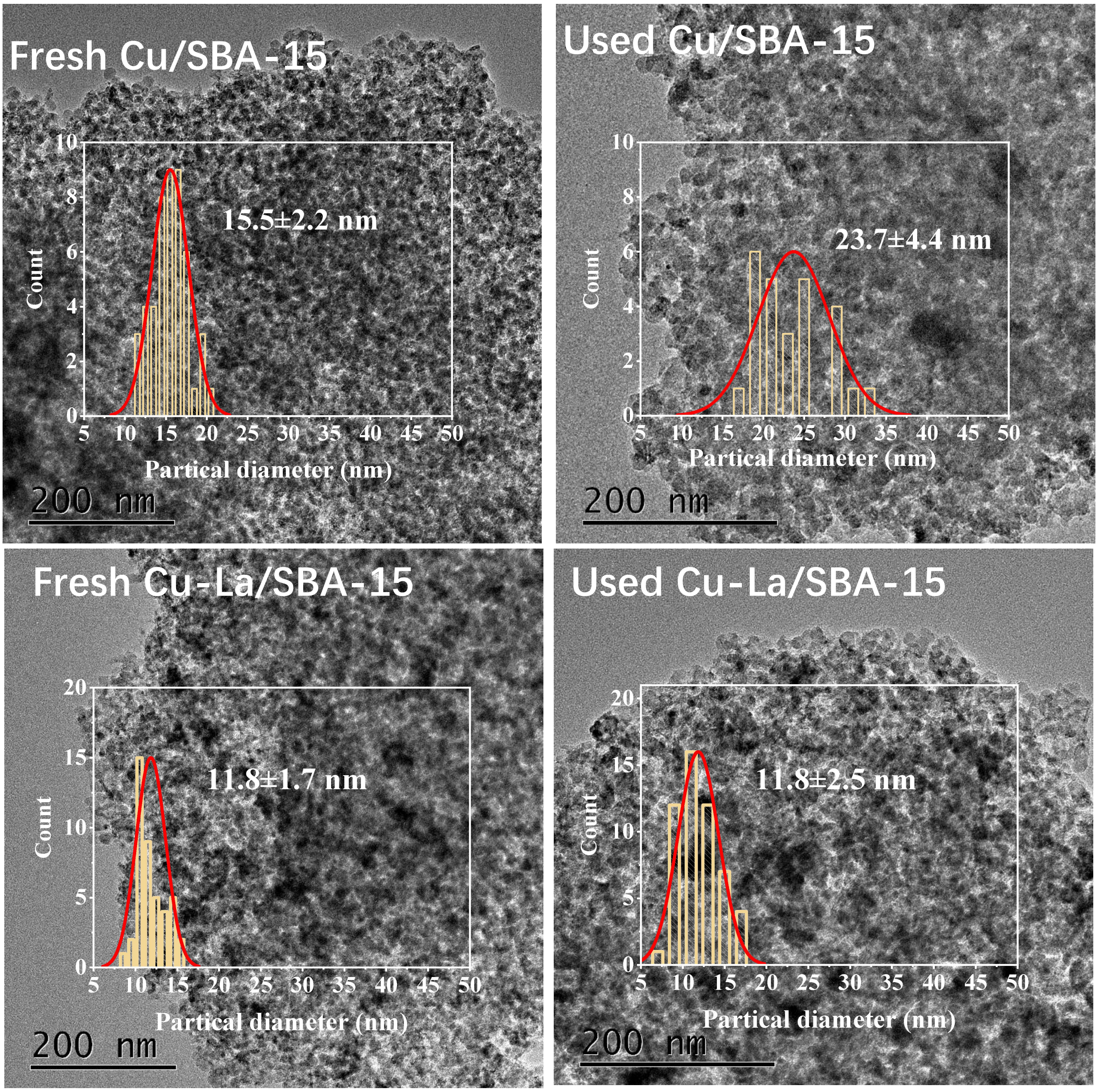

Supplementary Fig. 11 TEM images of the fresh and used Cu/SBA-15 and Cu-La/SBA-15 catalysts.

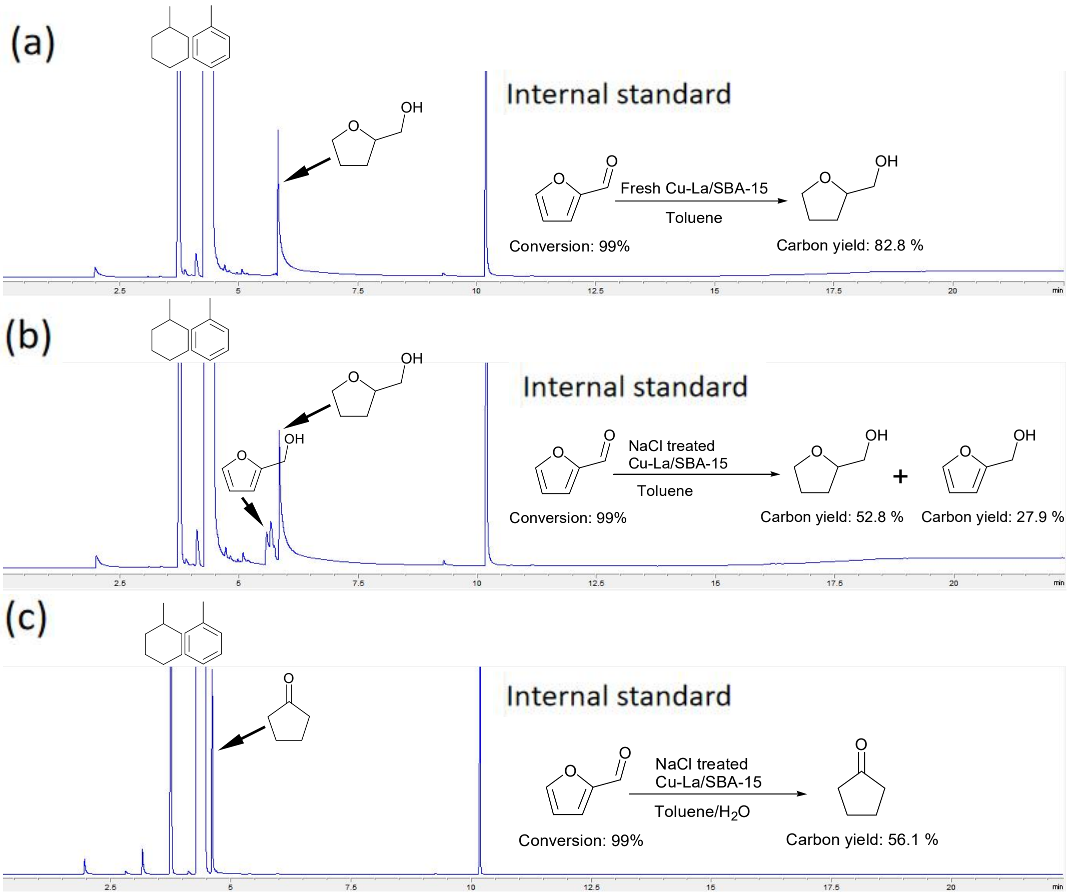

Supplementary Fig. 12 GC chromatograms of the products obtained from the hydrogenation of furfural over the (a) fresh and (b, c) NaCl-treated Cu-La/SBA-15 catalysts in toluene. Reaction conditions: (a, b) 3 MPa H_2, 433 K, 4 h, 400 rpm; 20 mL toluene, 0.5 g furfural and 25 mg Cu-La/SBA-15 catalyst were used for the tests. (c) 3 MPa H_2, 433 K, 4 h, 400 rpm; 20 mL toluene, 20 mL deionized water, 0.5 g furfural and 25 mg Cu-La/SBA-15 catalyst were used for the tests.

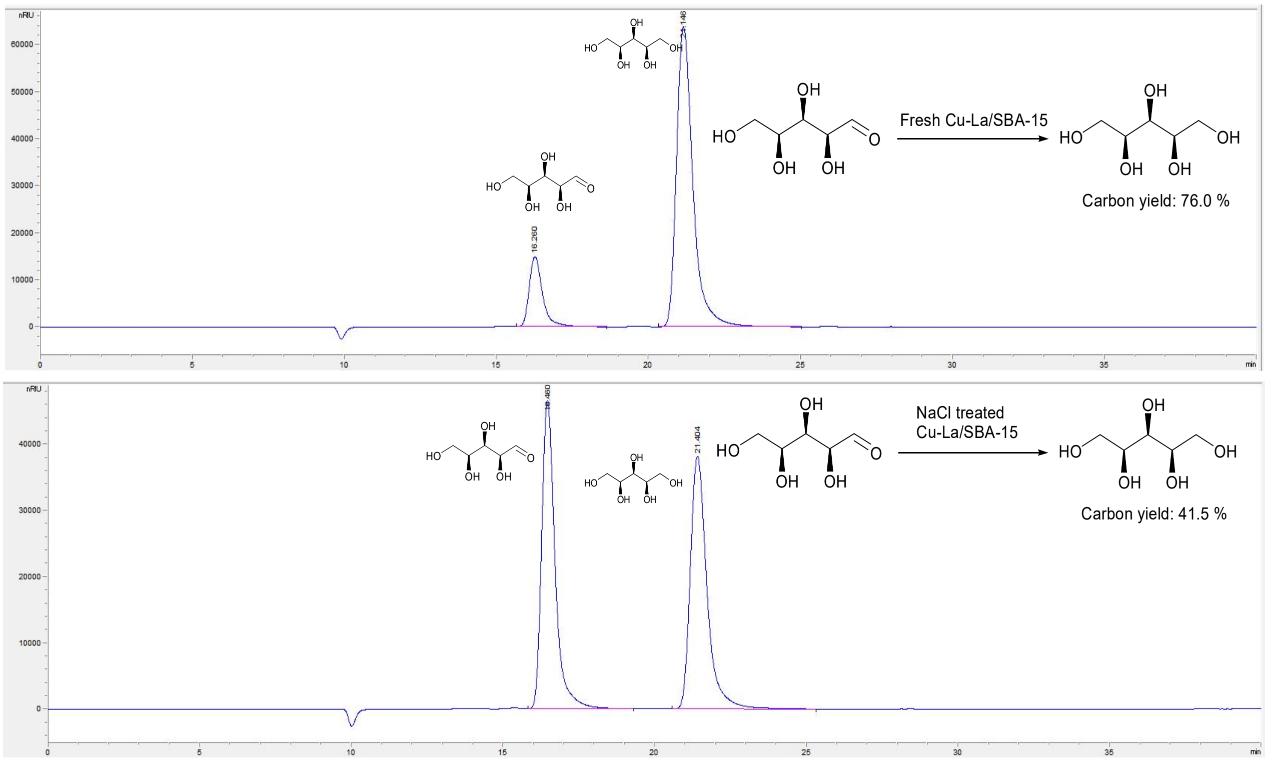

Supplementary Fig. 13 HPLC chromatograms of the products obtained from the hydrogenation of xylose over the fresh and NaCl-treated Cu-La/SBA-15 catalysts. Reaction conditions: 3 MPa H_2, 378 K, 500 rpm, 4 h; 10 deionized water, 0.3 g xylose and 30 mg fresh or NaCl treated Cu-La/SBA-15 catalyst were used for the test.

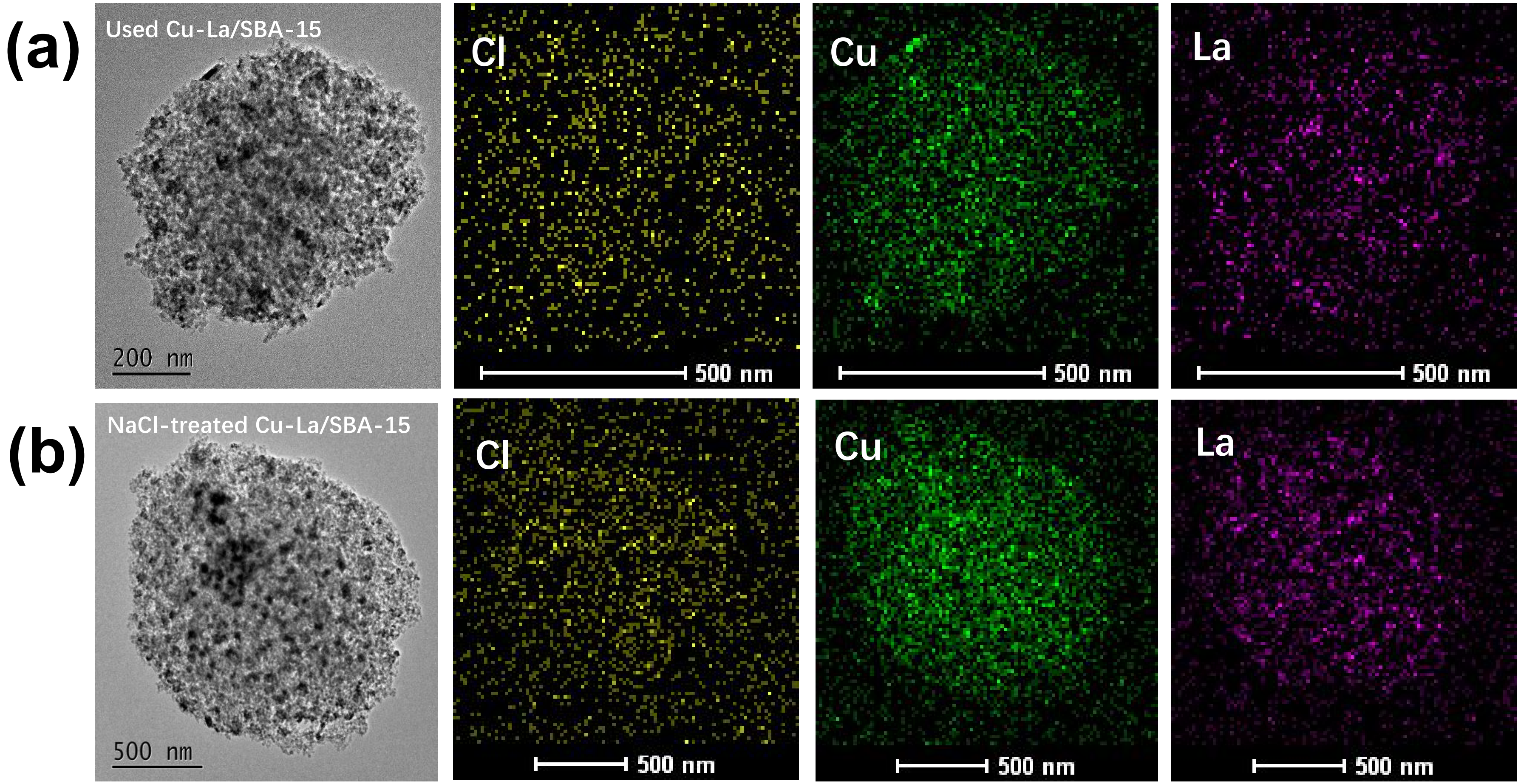

Supplementary Fig. 14 TEM-EDX elemental mappings results of (a) the used and (b) the NaCl treated Cu-La/SBA-15 catalysts.

According to TEM-EDX elemental mappings results, the metal particles on the surface of Cu-La/SBA-15 catalyst were partially covered by Cl species during the NaCl treatment or the usage in a toluene/NaCl aqueous solution biphasic reaction system, which may restrain the planar adsorption and hydrogenation of furan ring or the hydrogenation of xylose. This may be the reason for the restraining effect of NaCl on the over hydrogenation over the Cu-La/SBA-15 catalyst.

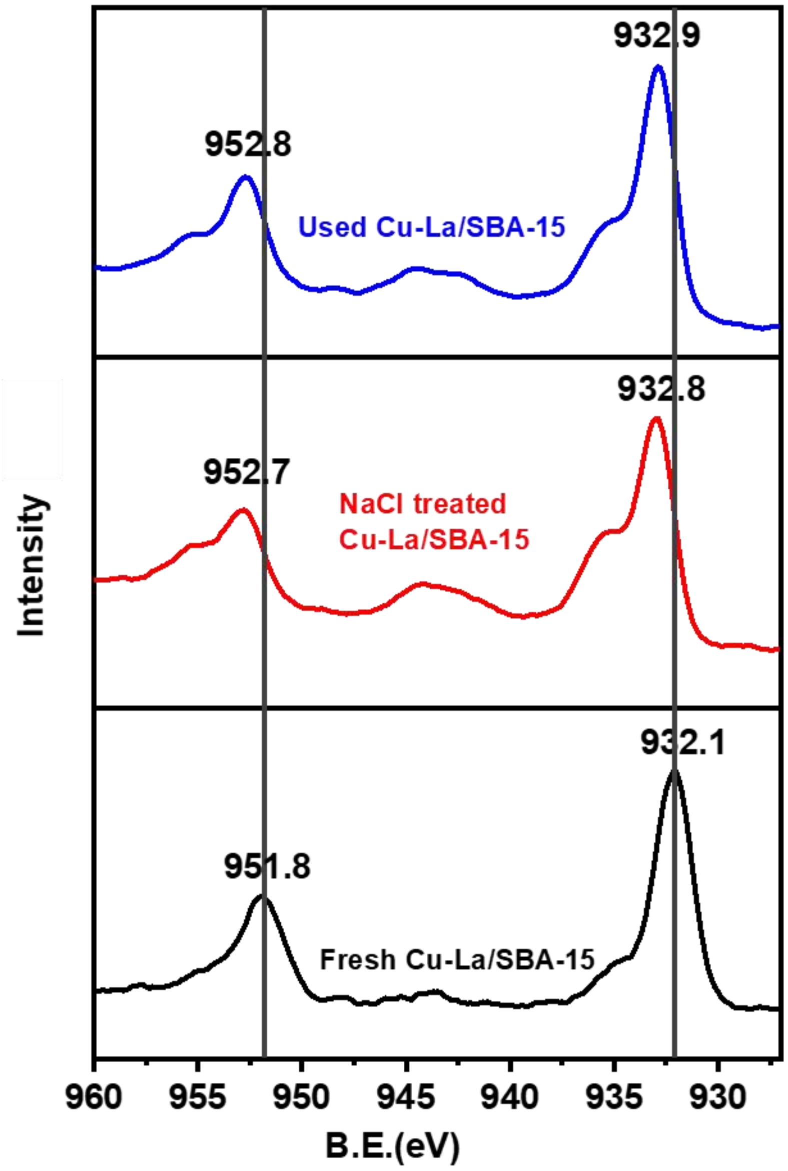

Supplementary Fig. 15 Cu *2p* XPS spectra of the fresh, NaCl treated and used Cu-La/SBA-15 catalysts.

From the XPS results illustrated in Supplementary Fig. 14, it is also noticed that the Cu *2p* band energy of the Cu-La/SBA-15 catalyst shifted to higher values after it was treated by the NaCl aqueous solution or used for the hydrogenolysis of xylose in the toluene/NaCl aqueous solution biphasic system which can be rationalized by the electron transfer from Cu species to the Cl species adsorbed on metal particles.

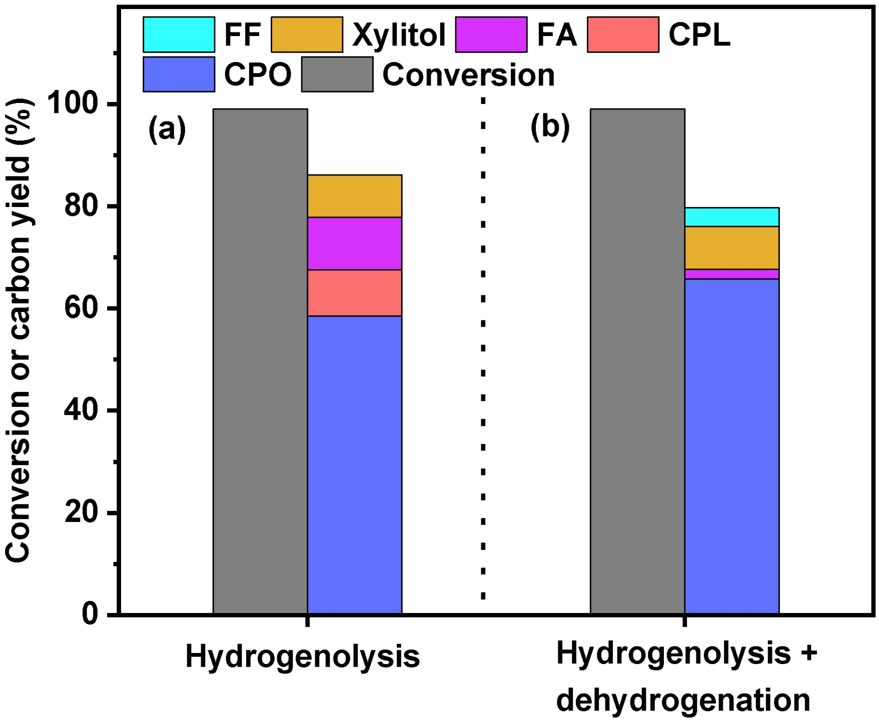

Supplementary Fig. 16 Xylose conversion and the carbon yields of different products from (a) hydrogenolysis of xylose over the Cu-La/SBA15 catalyst or (b) hydrogenolysis of xylose over the Cu-La/SBA15 catalyst and dehydrogenation of toluene phase product from the hydrogenolysis step over Pd/C catalyst. Reaction conditions: (a) 433 K, 3 MPa H_2, 500 rpm, 4 h; 10 mL 3 wt.% NaCl aqueous solution, 10 mL toluene, 0.3 g xylose and 30 mg catalyst were used for the test. (b) First step: 433 K, 3 MPa H_2, 500 rpm, 4 h; 10 mL 3 wt.% NaCl aqueous solution, 10 mL toluene, 0.3 g xylose and 30 mg catalyst were used for the test. Second step: 463 K, 1 MPa N_2, 500 rpm, 4 h, 10 mL toluene phase product from the first step and 20 mg Pd/C were used for the test. CPO: cyclopentanone. CPL: cyclopentanol. FA: furfuryl alcohol. FF: furfural.

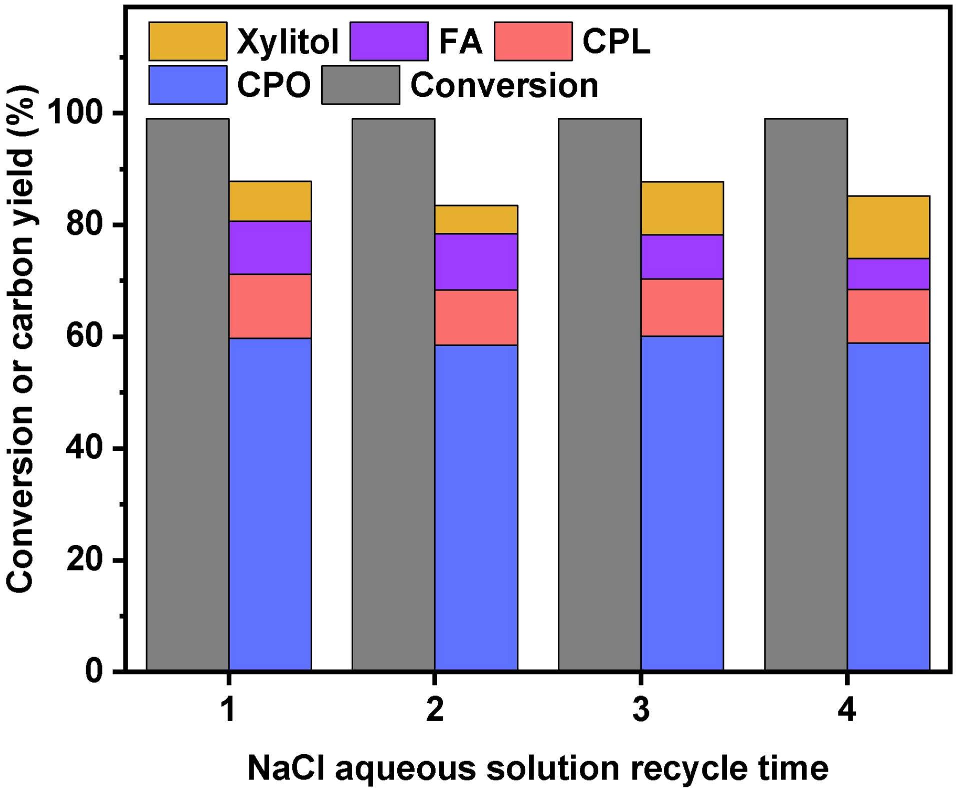

Supplementary Fig. 17 Xylose conversion and the carbon yields of different products from hydrogenolysis of xylose over the Cu-La/SBA-15 catalyst as the function of recycle time of NaCl aqueous solution. Reaction conditions: 433 K, 3 MPa H_2, 500 rpm, 4 h; 10 mL toluene, 10 mL 3 wt.% NaCl aqueous solution, 0.3 g xylose and 30 mg Cu-La/SBA-15 catalyst were used for the test. CPO: cyclopentanone. CPL: cyclopentanol. FA: furfuryl alcohol.

For environmental protection, we also checked the reuseability of the NaCl aqueous solution. To do this, we separated the aqueous phase product (a mixture of water, NaCl and small amount of sorbitol) by a separatory funnel and reused it as the NaCl aqueous solution in the next batch reaction. As we can see from Supplementary Fig. 17, the NaCl aqueous solution can be repeatedly used without any treatment, which is advantageous in real application.

Supplementary Table 2. The sugar concentrations in the hemicellulose aqueous solutions extracted from poplar wood or corncob (denoted as $\text{Hemicellulose}_{\text{poplar wood}}$ and $\text{Hemicellulose}_{\text{corncob}}$, respectively).

	Concentration (g L^{-1})		
	Total sugar	Xylose	Arabinose
$\text{Hemicellulose}_{\text{poplar wood}}$	10.7	9.1	1.6
$\text{Hemicellulose}_{\text{corncob}}$	11.2	9.5	1.7

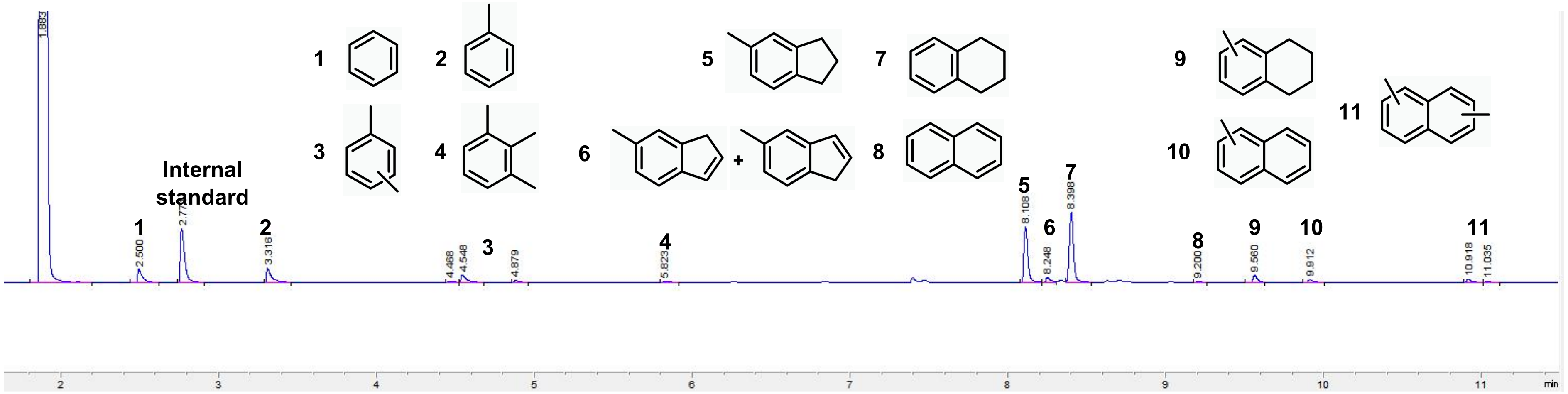

Supplementary Fig. 18 GC chromatogram of the products obtained from the reaction of CPO over the H-ZSM-5 (160) catalyst. Reaction conditions: 0.1 MPa N_2, 723 K, WHSV = 0.45 g g^{-1} h^{-1}, the initial N_2/CPO molar ratio = 36/1. CPO: cyclopentanone. WHSV: weight hour space velocity.

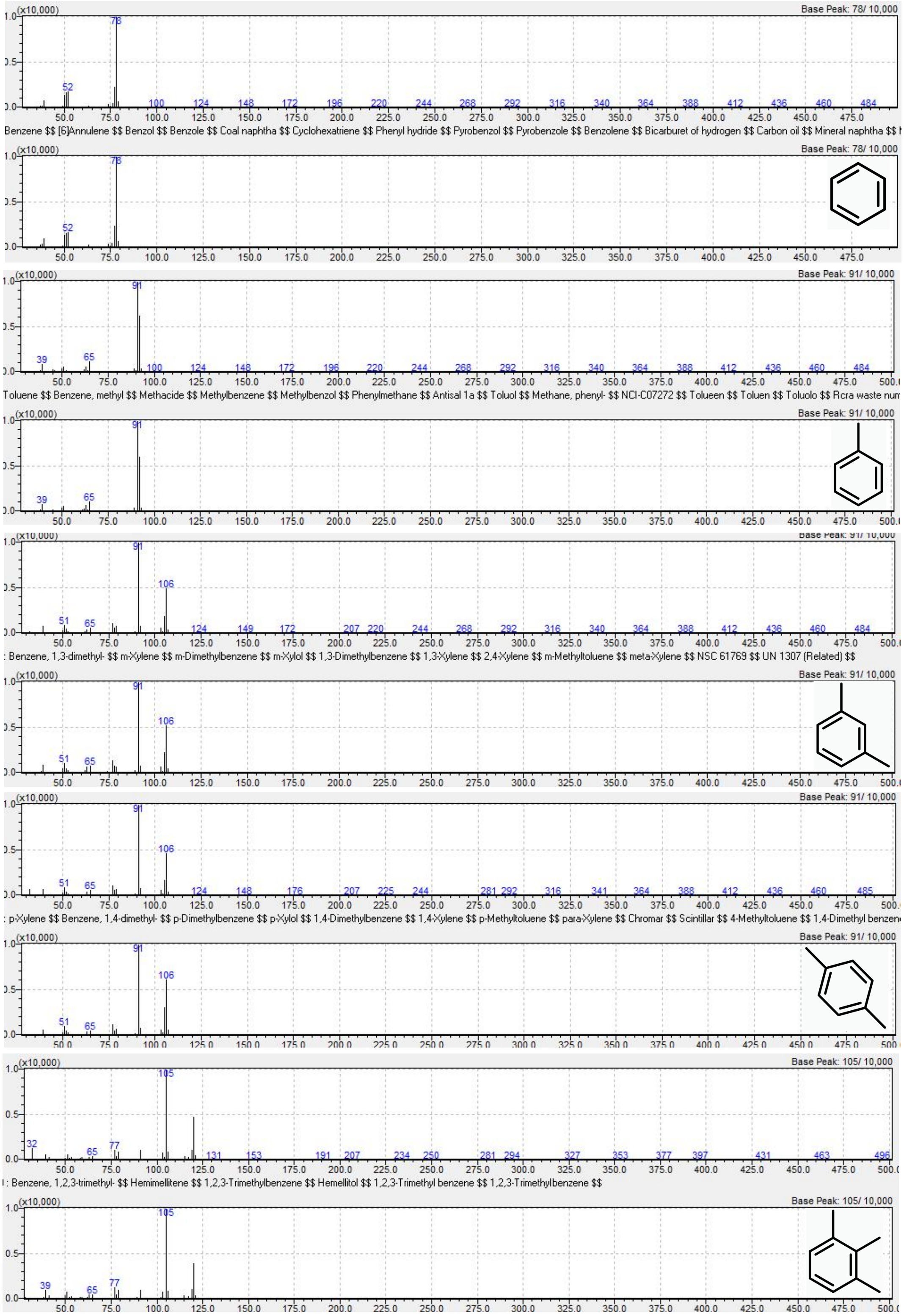

Supplementary Fig. 19 Mass spectrograms of the C_6-C_9 aromatics obtained from the reaction of CPO over the zeolite catalysts. Reaction conditions: 0.1 MPa N_2, 723 K, WHSV = 0.45 g g^{-1} h^{-1}, the initial N_2/CPO molar ratio = 36/1. CPO: cyclopentanone. WHSV: weight hour space velocity.

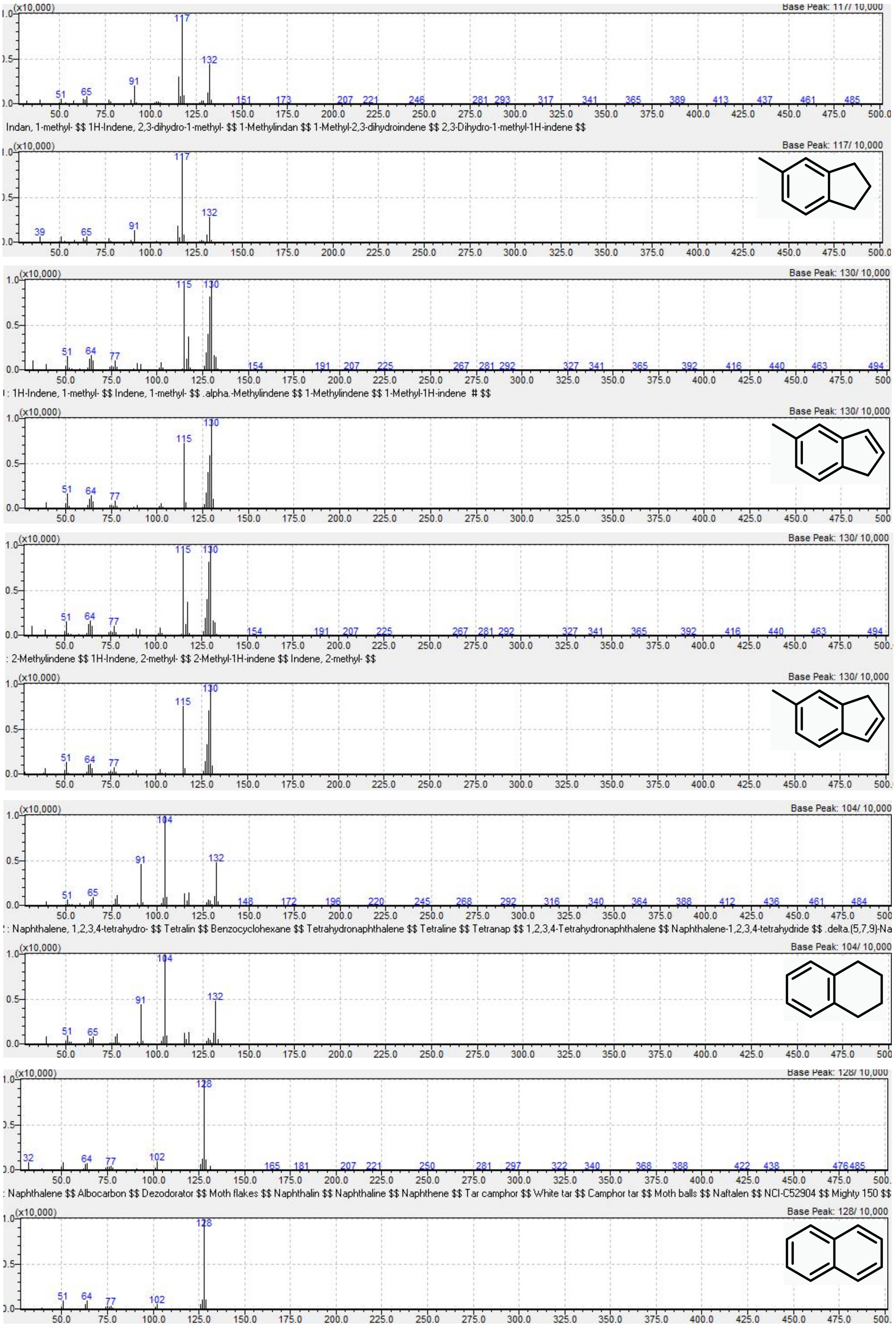

Supplementary Fig. 20 Mass spectrograms of the C_{10} aromatics obtained from the reaction of CPO over the zeolite catalysts. Reaction conditions: 0.1 MPa N_2, 723 K, WHSV = 0.45 g g^{-1} h^{-1}, the initial N_2/CPO molar ratio = 36/1. CPO: cyclopentanone. WHSV: weight hour space velocity.

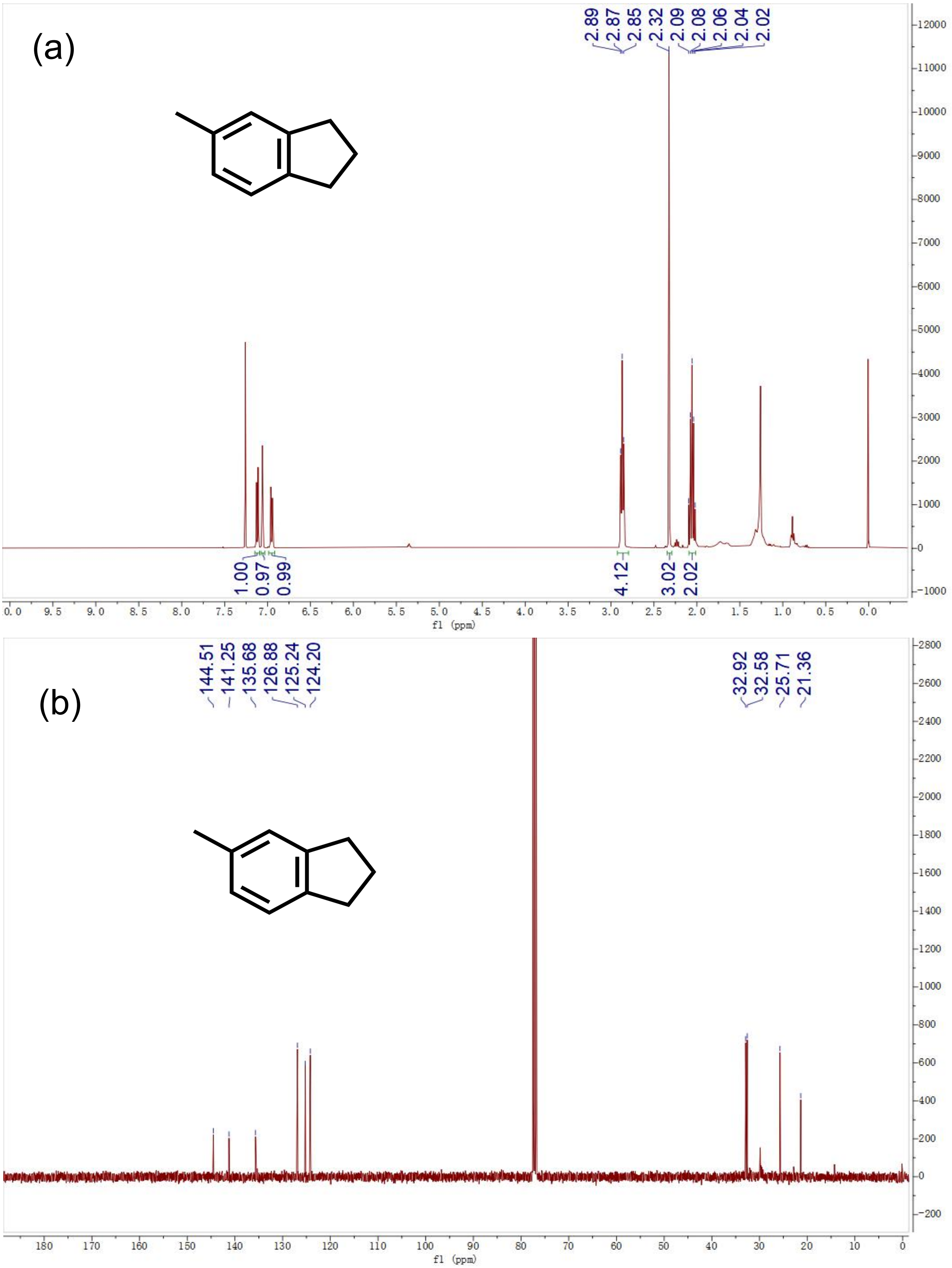

Supplementary Fig. 21 ^{1}H NMR (a) and ^{13}C NMR (b) spectra of the 5-methyl-2,3-dihydro-1*H*-indene (abbreviated as methylindan) obtained from the reaction of CPO over the H-ZSM-5 (160) catalyst. CPO: cyclopentanone.

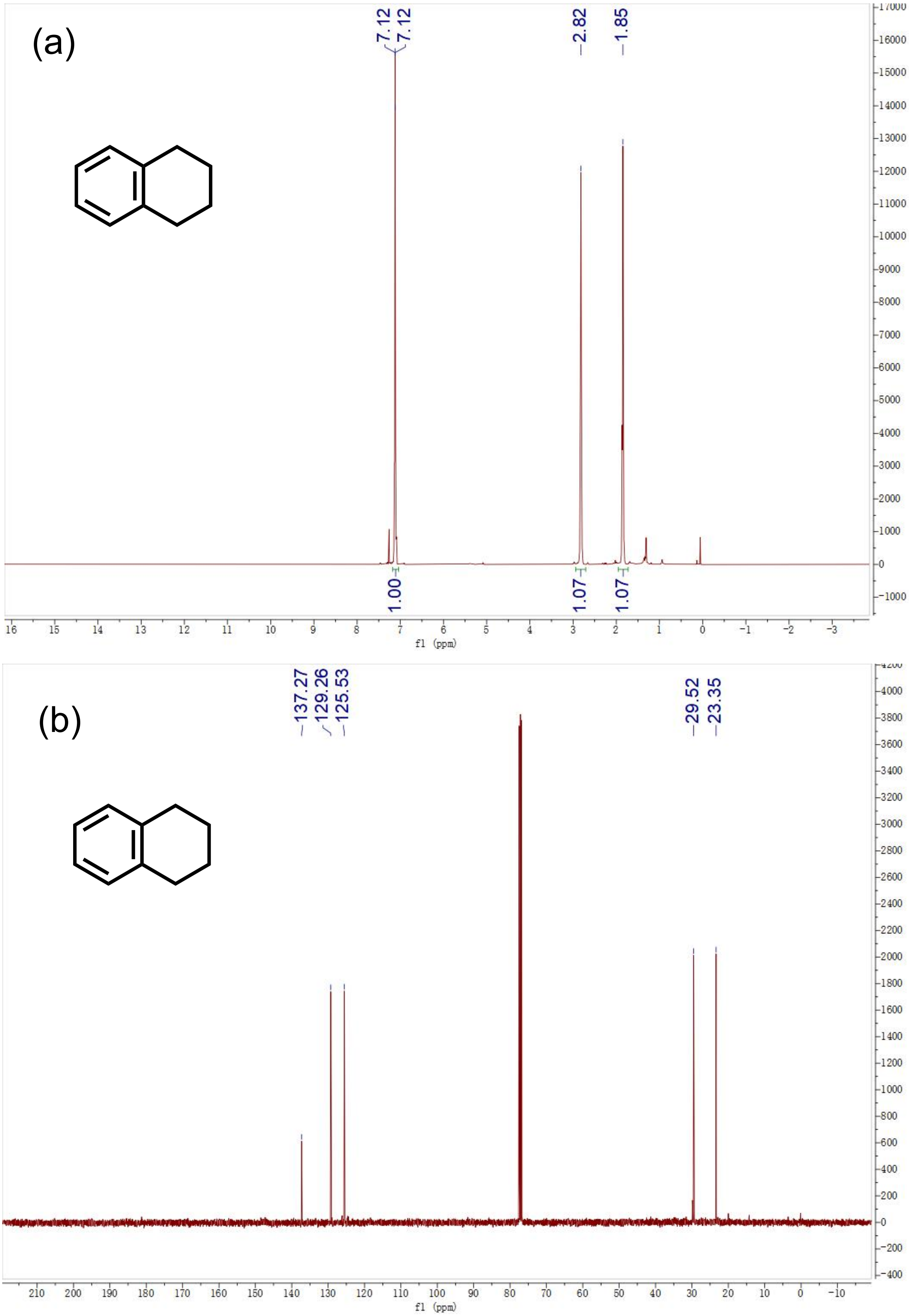

Supplementary Fig. 22 ^{1}H NMR (a) and ^{13}C NMR (b) spectra of the tetralin obtained from the reaction of CPO over the H-ZSM-5 (160) catalyst. CPO: cyclopentanone.

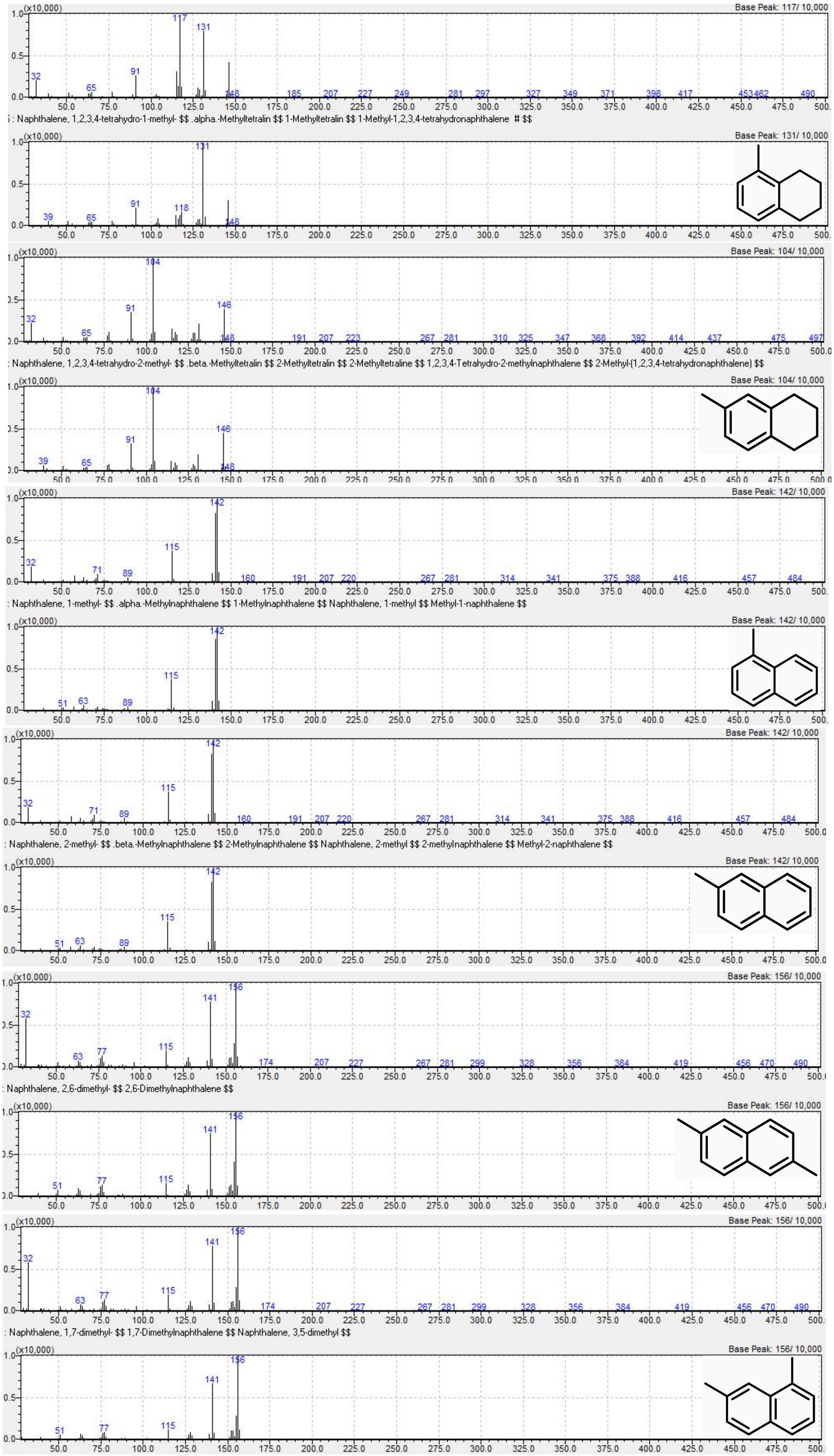

Supplementary Fig. 23 Mass spectrograms of the C_{11}-C_{12} aromatics obtained from the reaction of CPO over the zeolite catalysts. Reaction conditions: 0.1 MPa N_2, 723 K, WHSV = 0.45 g g^{-1} h^{-1}, the initial N_2/CPO molar ratio = 36/1. CPO: cyclopentanone. WHSV: weight hour space velocity.

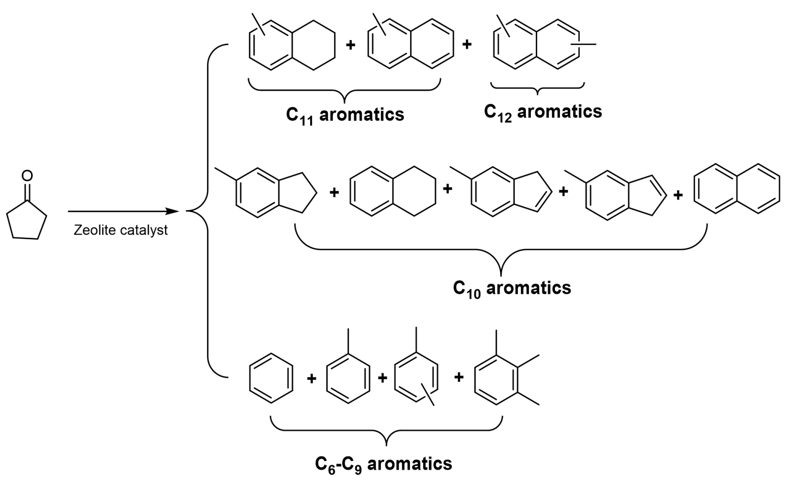

Supplementary Fig. 24 Reaction pathways for the production of various aromatics from CPO over zeolite catalysts. CPO: cyclopentanone.

Supplementary Table 3. The specific BET surface areas (S_{BET}) and the amount of acid sites of the zeolite catalysts used in this work.

	S_{BET} (m^2 g^{-1})[a]	Amount of acid sites (μmol g^{-1})[b]
SAPO-34	502	1418
H-Y	668	911
H-USY	666	1100
H-β	461	565
H-ZSM-5 (100)	338	493

[a] Measured by N_2-physisorption. [b] Measured by NH_3-chemisorption.

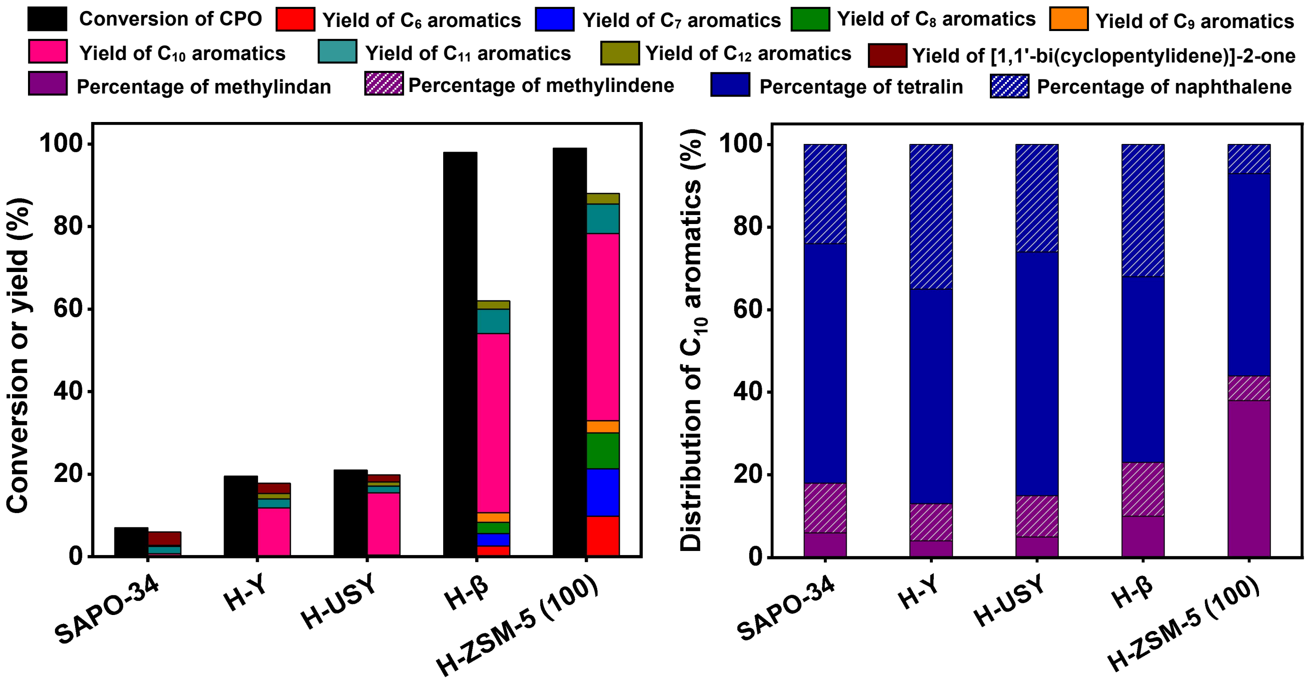

Supplementary Fig. 25 CPO conversion, the carbon yields of various products and the distribution of C_{10} aromatics over the zeolite catalysts with the fixed amount of acid sites (300 μmol). Reaction conditions: 723 K, the feed rate of CPO was 0.27 g h^{-1}, the amounts of catalysts were calculated according to the NH_3-chemisorption results (Supplementary Table 3), 0.1 MPa N_2, the initial N_2/CPO molar ratio = 36/1. CPO: cyclopentanone.

Supplementary Table 4. Specific BET surface area (S_{BET}) and the acid properties of the H-ZSM-5 catalysts used in this work.

	S_{BET} (m^2 g^{-1})[a]	Amount of acid sites (μmol g^{-1})			Strong acid sites / weak acid sites ratio[b]	Brönsted acid sites / Lewis acid sites ratio[d]	
		Weak acid sites[b]	Strong acid sites[b]	Total acid sites[c]		423 K[e]	623 K[e]
H-ZSM-5 (21)	314	706	575	1281	0.815	3.180	3.027
H-ZSM-5 (60)	288	416	468	884	1.127	5.796	5.116
H-ZSM-5 (100)	338	232	261	493	1.126	2.768	2.999
H-ZSM-5 (160)	367	175	211	386	1.204	1.341	1.732
H-ZSM-5 (200)	344	198	183	381	0.919	2.818	3.002

[a] Measured by N_2-physisorption. [b] Measured by NH_3-TPD. [c] Measured by NH_3-chemisorption. [d] Measured by FT-IR using pyridine as a probe molecule. [e] Desorption temperature.

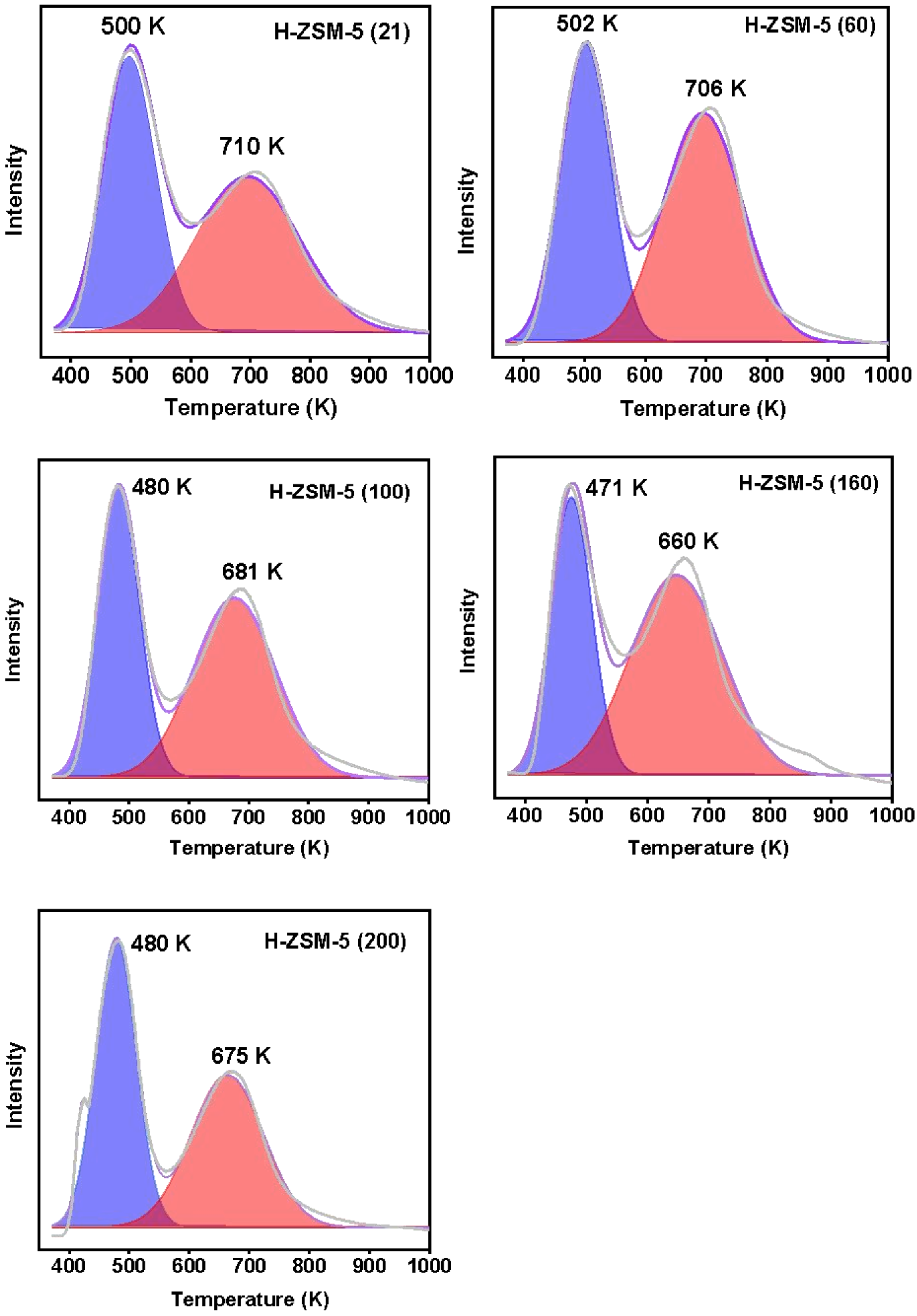

Supplementary Fig. 26 NH_3-TPD profiles of the H-ZSM-5 catalysts used in this work.

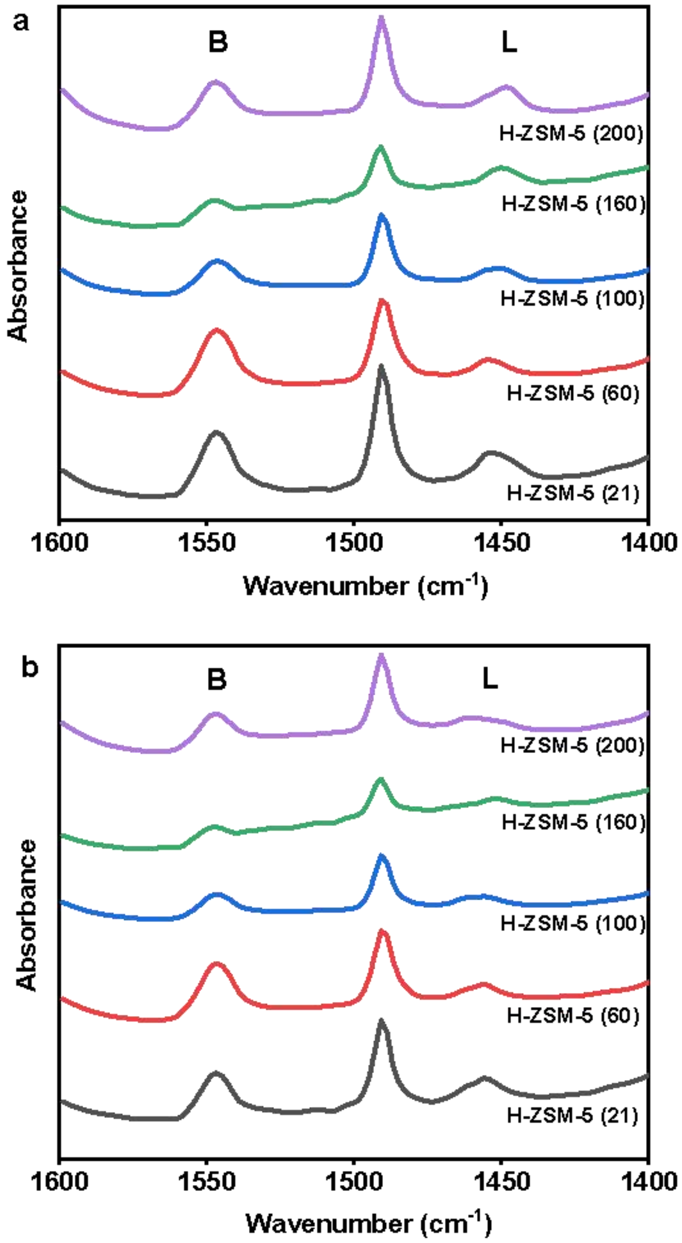

Supplementary Fig. 27 FT-IR spectra of H-ZSM-5 catalysts after the adsorption of pyridine at room temperature and the desorption in vacuum at (a) 423 K and (b) 623 K. B: Brönsted acid sites; L: Lewis acid sites.

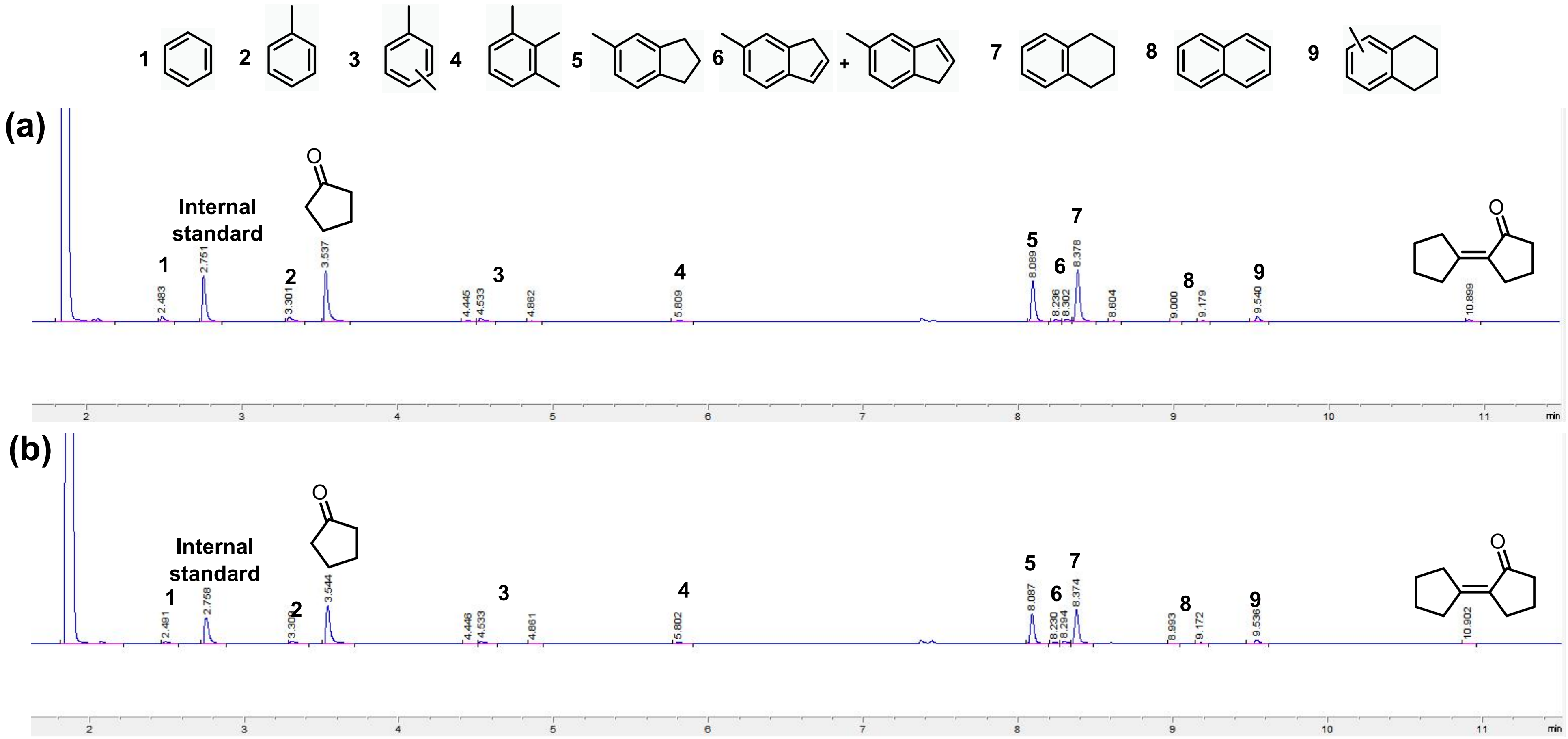

Supplementary Fig. 28 GC chromatograms of the products that were obtained from the reaction of CPO over the H-ZSM-5 (160) catalyst at a relatively low reaction temperature (a) or a relatively high WHSV (b). Reaction conditions: (**a**) 0.1 MPa N_2, 623 K, WHSV = 0.45 g g^{-1} h^{-1}, the initial N_2/CPO molar ratio = 36/1. (**b**) 0.1 MPa N_2, 698 K, WHSV = 1.2 g g^{-1} h^{-1}, the initial N_2/CPO molar ratio = 36/1. CPO: cyclopentanone. WHSV: weight hour space velocity.

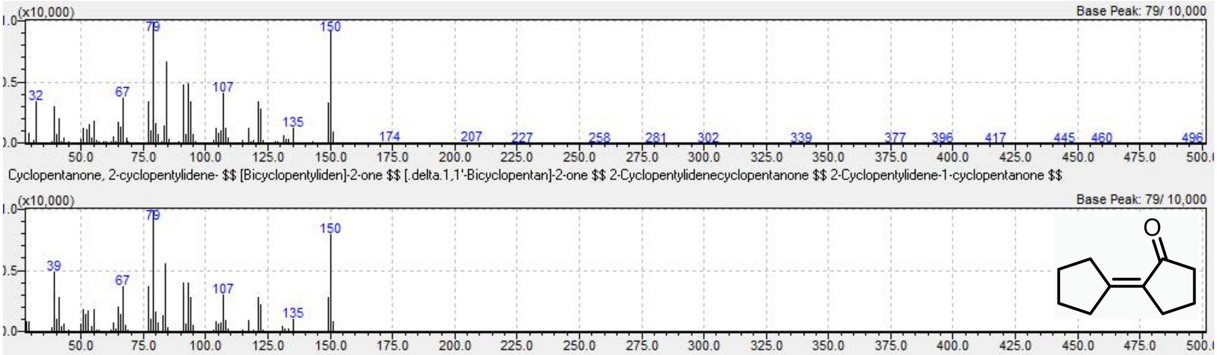

Supplementary Fig. 29 Mass spectrogram of the [1,1'-bi(cyclopentylidene)]-2-one obtained from the reaction of CPO over the H-ZSM-5 (160) catalyst. CPO: cyclopentanone.

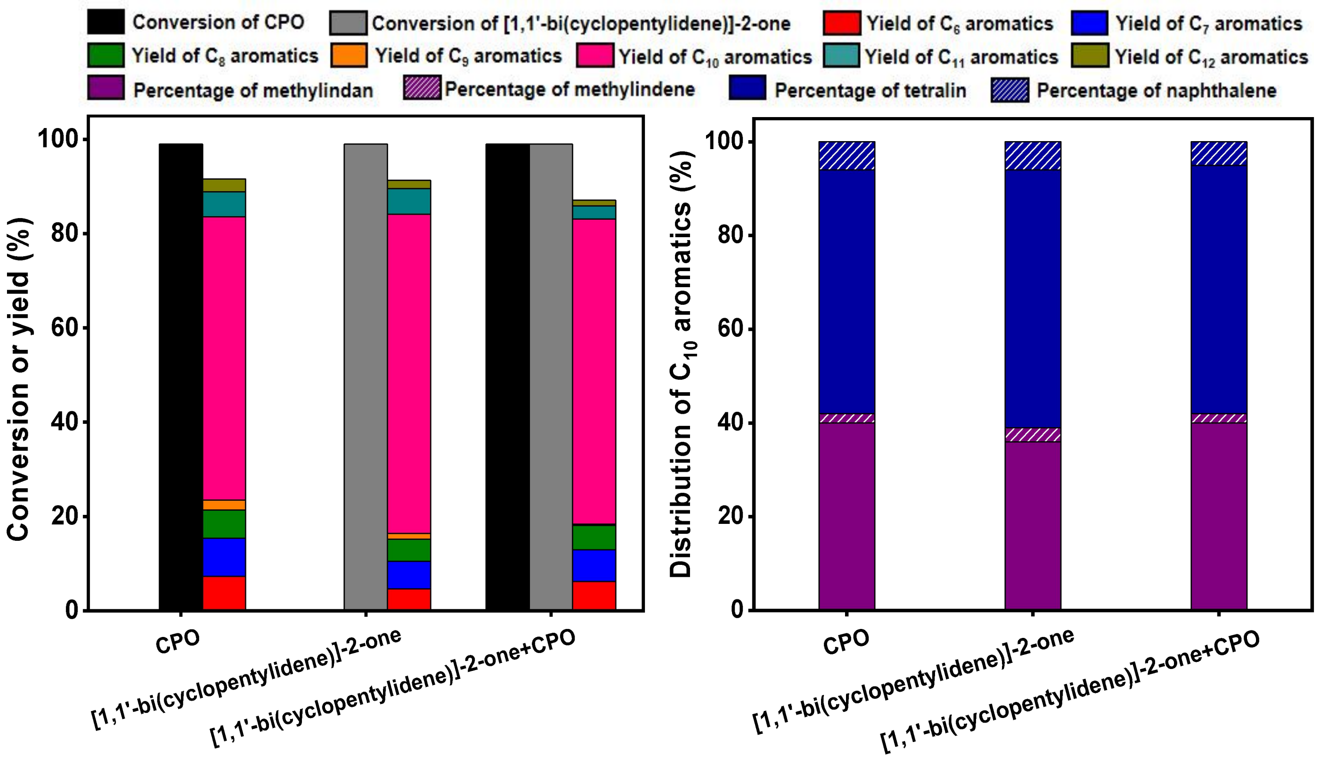

Supplementary Fig. 30 Conversions of CPO or [1,1'-bi(cyclopentylidene)]-2-one, the carbon yields of products and the distributions of C_{10} aromatics (including methylindan, methylindene, tetralin and naphthalene) over the H-ZSM-5 (160) catalyst. Reaction conditions: 0.1 MPa N_2, 723 K, WHSV = 0.45 g g^{-1} h^{-1}, the initial N_2/substrates molar ratio = 36/1. CPO: cyclopentanone. WHSV: weight hour space velocity.

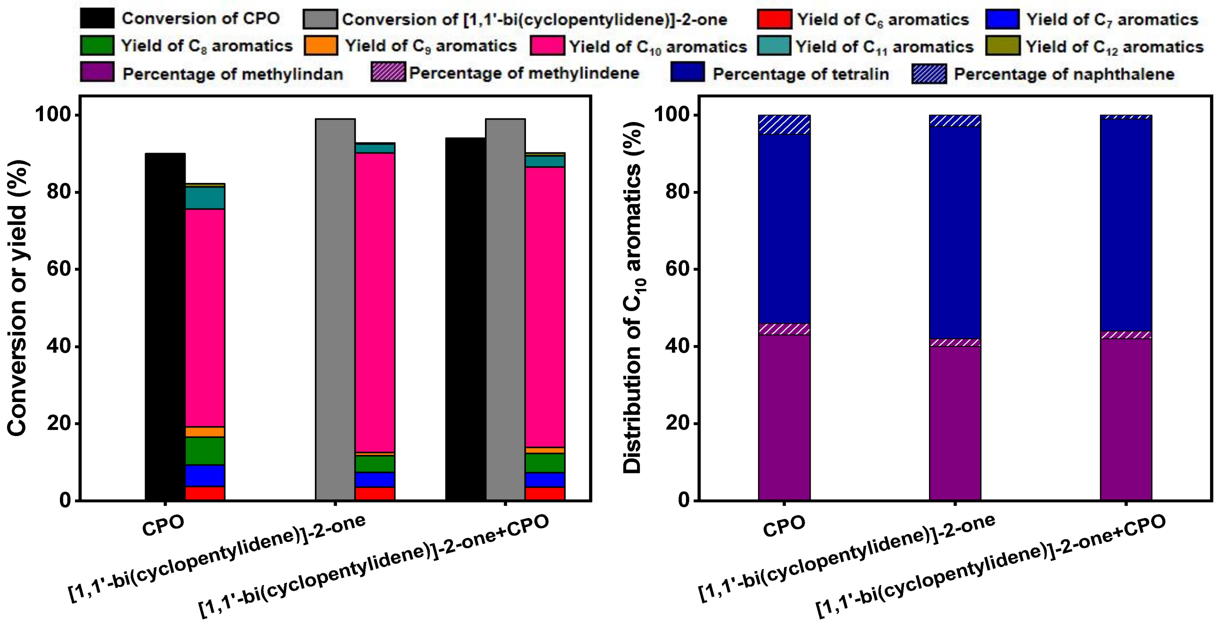

Supplementary Fig. 31 Conversions of CPO or [1,1'-bi(cyclopentylidene)]-2-one, the carbon yields of products and the distributions of C_{10} aromatics (including methylindan, methylindene, tetralin and naphthalene) over the H-ZSM-5 catalysts. Reaction conditions: 0.1 MPa N_2, 673 K, WHSV = 0.45 g g^{-1} h^{-1}, the initial N_2/substrate molar ratio = 36/1. CPO: cyclopentanone. WHSV: weight hour space velocity.

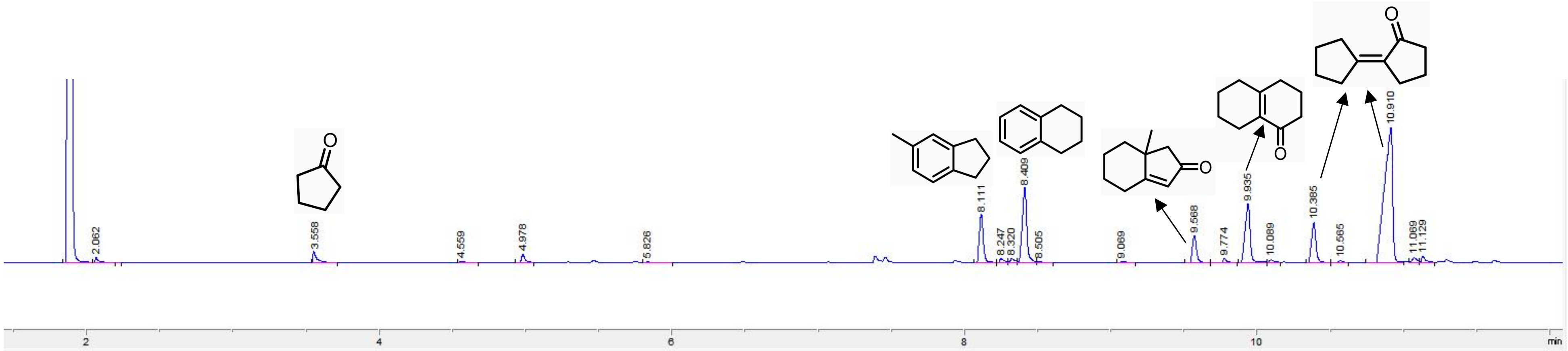

Supplementary Fig. 32 GC chromatogram of the products obtained from the reaction of [1,1'-bi(cyclopentylidene)]-2-one over the H-ZSM-5 (160) catalyst at a relatively low reaction temperature (623 K). Reaction conditions: 0.1 MPa N_2, 623 K, WHSV = 0.6 g g^{-1} h^{-1}, the initial N_2/[1,1'-bi(cyclopentylidene)]-2-one molar ratio = 36/1. WHSV: weight hour space velocity.

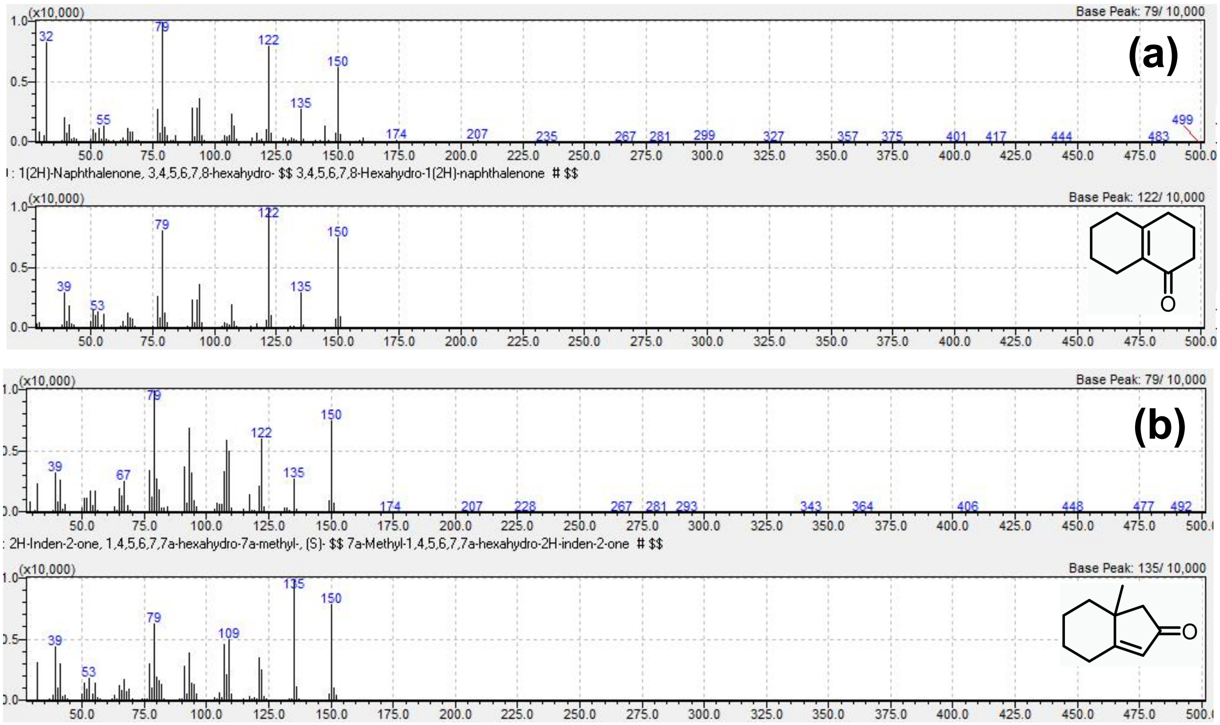

Supplementary Fig. 33 Mass spectrograms of the 3,4,5,6,7,8-hexahydronaphthalen-1(2*H*)-one (HHNO) (a) and 7a-methyl-5,6,7,7a-tetrahydro-1*H*-inden-2(4*H*)-one (MTHIO) (b) obtained from the reaction of [1,1'-bi(cyclopentylidene)]-2-one over the H-ZSM-5 (160) catalyst at a relatively low reaction temperature (623 K).

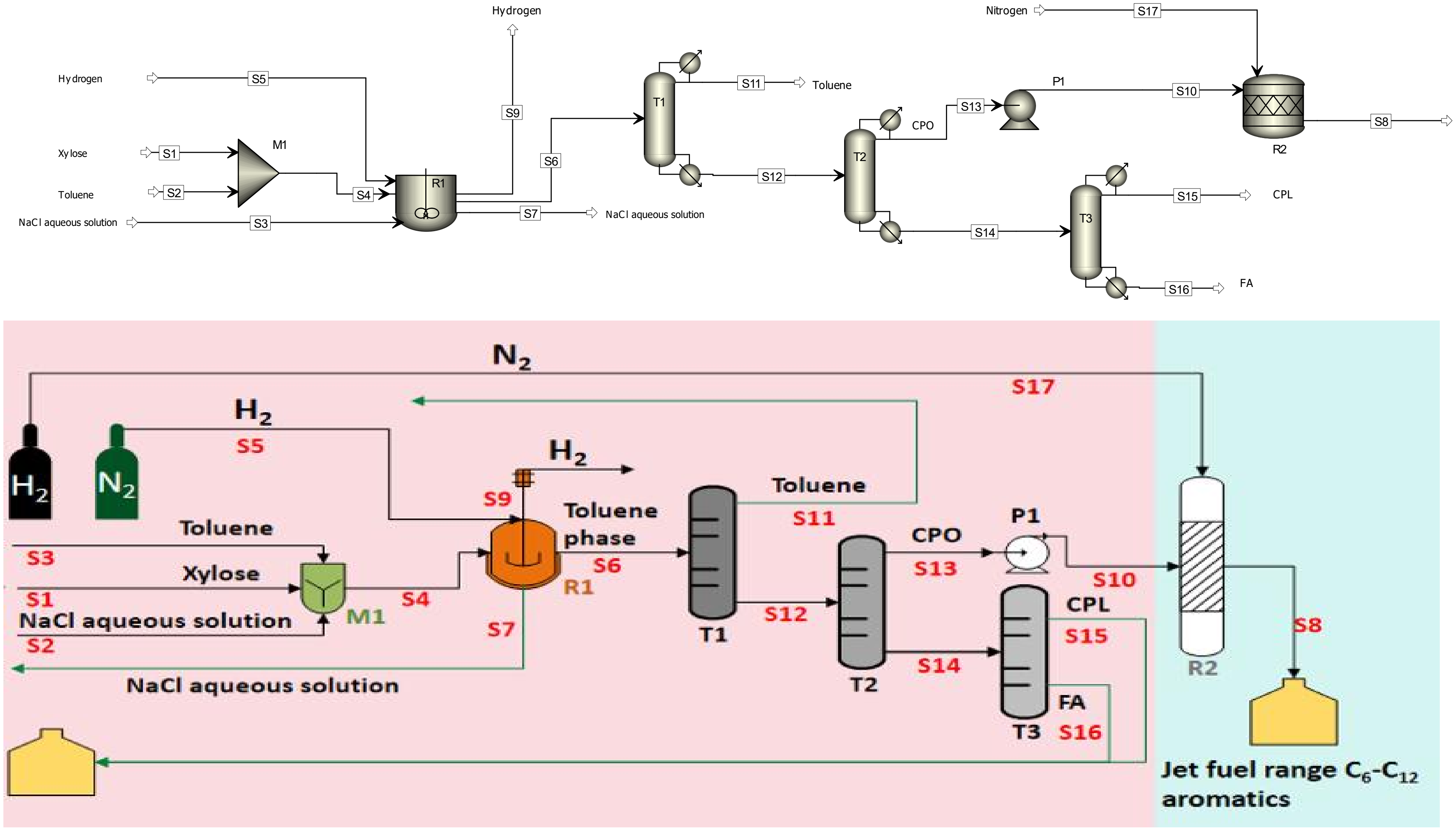

Supplementary Fig. 34 Process flow diagram for the conversion of xylose to methylindan and tetralin under the conditions of 50 L reactor. CPO: cyclopentanone. CPL: cyclopentanol. FA: furfuryl alcohol.

Supplementary Table 5. Material balance calculation for the conversion of xylose in a 50 L reactor.[a]

Components	Reactant (Kg h^{-1})	Products (Kg h^{-1})
Xylose	1080.0	
H_2	36.3	
Methylindan + tetralin		172.0
Methylindene		5.2
Naphthalene		3.7
C_6-C_9 aromatics		34.4
C_{11}-C_{12} aromatics		13.8
Xylitol		42.7
FA		69.8
CPL		96.7
Water		470.3
Sum	1116.3	908.6

[a] The entire designed process was simulated for steady-state material and energy flow using Aspen Plus V11.

The Rstoic block was employed to simulate the reactor. The product distribution was compared with experimental data. The RadFrac module was utilized to simulate the distillation column equipment. The Mixer module replicated the fluid blending process. The Pump module simulated the fluid transport pump process. CPL: cyclopentanol. FA: furfuryl alcohol.

Supplementary Table 6. The material balance calculation during the chemical process simulation for conversion of xylose under the conditions of a 50 L reactor.[a]

Module	Component	Feeding (Kg h^{-1})	Discharge (Kg h^{-1})	
M1	Xylose	1080.0	1080.0	
	NaCl	1103.4	1103.4	
	Water	35675.2	35675.2	
M1 Sum.		37858.6	37858.6	
R1	Xylose	1080.0		
	NaCl	1103.4	1103.4	
	Water	35675.2	36096.2	
	Toluene	31129.4	31129.4	
	CPO		352.2	
	FA		69.8	
	CPL		96.7	
	Xylitol		42.7	
R1 Sum.		68988.0	68890.4	
S6	Toluene	31129.4	31129.4	
	CPO	352.2	352.2	
	FA	69.8	69.8	
	CPL	96.7	96.7	
S6 Sum.		31648.1	31648.1	
S7	NaCl	1103.4	1103.4	
	Water	36096.2	36096.2	
	Xylitol	42.7	42.7	
S7 Sum.		37242.3	37242.3	
T1			Top light components	Bottom heavy components
	Toluene	31129.4	31118.8	10.7
	CPO	352.2		341.4
	FA	69.8		69.8
	CPL	96.7		96.7
T1 Sum.		31648.1	31637.4	
T2			Top light components	Bottom heavy components
	Toluene	10.7	10.7	1.27×10^{-5}
	CPO	341.4	338.2	3.2
	FA	69.8	1.60×10^{-5}	69.9

	CPL	96.7	3.4	93.2
T2 Sum.		518.6	518.6	
T3			Top light components	Bottom heavy components
	Toluene	1.27×10^{-5}	1.27×10^{-5}	1.58×10^{-13}
	CPO	3.2	3.2	2.16×10^{-5}
	FA	69.9	0.02	69.8
	CPL	93.2	93.2	0.02
T3 Sum.		166.3	166.2	
P1	Toluene	10.7	10.7	
	CPO	338.2	338.2	
	FA	1.60×10^{-5}	1.60×10^{-5}	
	CPL	3.4	3.4	
P1 Sum.		352.3	352.3	
R2	Toluene	10.7	10.7	
	CPO	338.2		
	FA	1.60×10^{-5}	1.60×10^{-5}	
	CPL	3.4	3.4	
	Methylindan		77.9	
	Tetralin		94.1	
	Naphthalene		5.2	
	Methylindene		3.7	
	C_6-C_9 aromatics		34.4	
	C_{11}-C_{12} aromatics		13.8	
	Water		49.3	
R2 Sum.		352.3	281.8	

[a] The entire designed process was simulated for steady-state material and energy flow using Aspen Plus V11

The Rstoic block was employed to simulate the reactor

The product distribution was compared with experimental data

The RadFrac module was utilized to simulate the distillation column equipment

The Mixer module replicated the fluid blending process

The Pump module simulated the fluid transport pump process

CPO: cyclopentanone. CPL: cyclopentanol. FA: furfuryl alcohol.

Supplementary Table 7. Energy balance calculation for the conversion of xylose in a 50 L reactor [a]

Pipeline	Feeding (KJ)	Discharge (KJ)
s1	-7573045.2	
s2	-572609895.9	
s3	4134498.9	
s5	2.71×10^{-10}	
s17	0	
s7		-557334843.1
s8		1066895.3
s9		214684.6
s11		7714902.7
s15		-1045992.5
s16		-309481.0
Sum.	-576048442.2	-549693834

a The entire designed process was simulated for steady-state material and energy flow using Aspen Plus V11. The NRTL equation was selected as the thermodynamic property method.

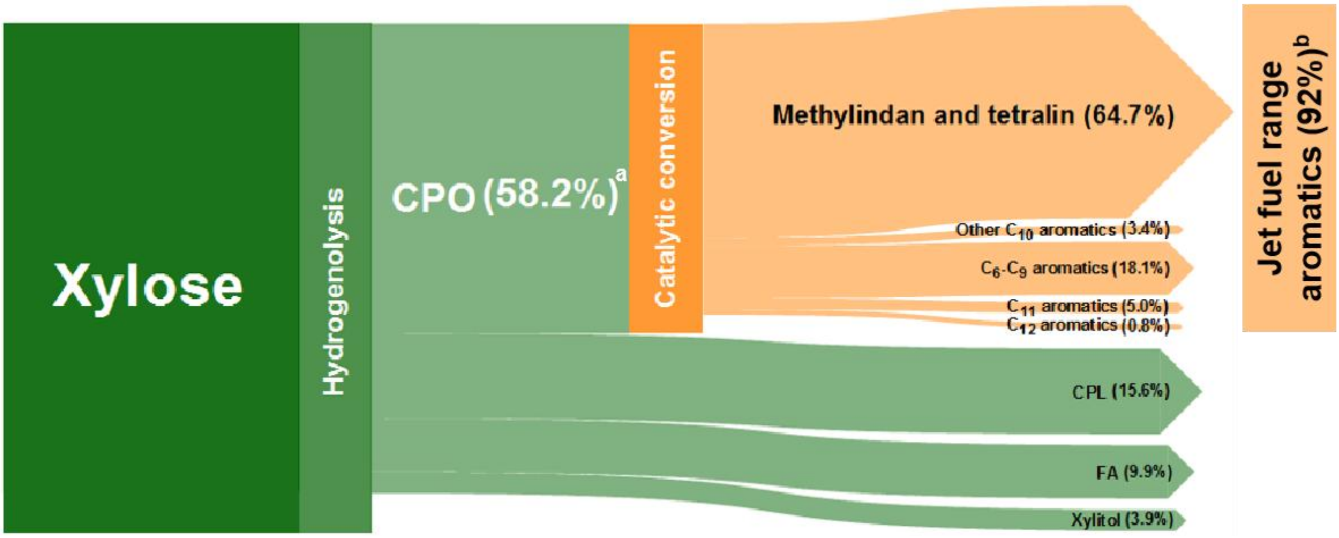

**Step 1**	**Substrate: xylose**	**Step 2**	**Substrate: CPO**
Reaction conditions	Cu-La/SBA-15, 433 K, 3 MPa H_2, 4 h, toluene/NaCl biphasic system.	Reaction conditions	H-ZSM-5 (160), WHSV = 0.6 g g^{-1} h^{-1}, 698 K, 0.1 MPa N_2, the initial N_2/CPO molar ratio = 36/1.
Conversion (%)	99		99
Products distribution (%)		Carbon distribution (%)	
CPO	58.2	C_6-C_9 aromatics	18.1
CPL	15.6	Methylindan and tetralin	64.7
FA	9.9	Other C_{10} aromatics	3.4
Xylitol	3.9	C_{11} aromatics	5.0
		C_{12} aromatics	0.8
Carbon balance (%)	87.6	Carbon balance (%)	92.0

Supplementary Fig. 35 Sankey diagram based on carbon balance in the conversion of xylose to different products. CPO: cyclopentanone. CPL: cyclopentanol. FA: furfuryl alcohol. WHSV: weight hour space velocity. [a] Carbon yield calculated based on xylose. [b] Carbon yield calculated based on CPO.

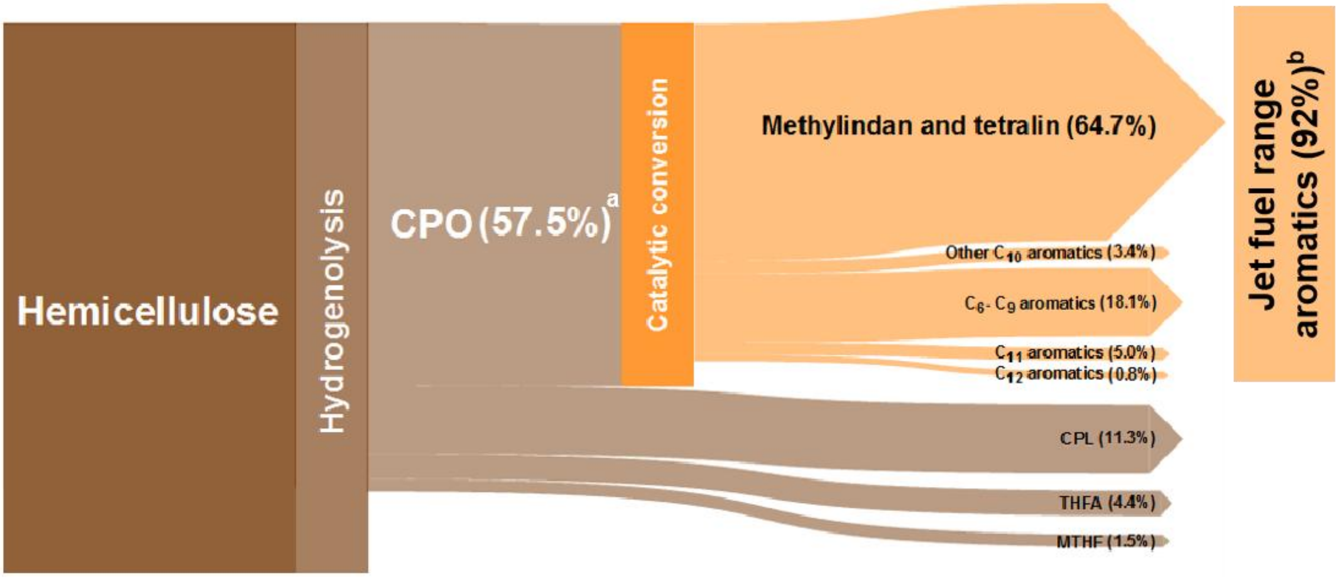

Step 1	Substrate: hemicellulose	Step 2	Substrate: CPO
Reaction conditions	Cu-La/SBA-15, 433 K, 3 MPa H_2, 4 h, toluene/NaCl biphasic system.	Reaction conditions	H-ZSM-5 (160), WHSV = 0.6 g g^{-1} h^{-1}, 698 K, 0.1 MPa N_2, the initial N_2/CPO molar ratio = 36/1.
Conversion (%)	99		99
Products distribution (%)		Carbon distribution (%)	
CPO	57.5	C_6-C_9 aromatics	18.1
CPL	11.3	Methylindan and tetralin	64.7
THFA	4.4	Other C_{10} aromatics	3.4
MTHF	1.5	C_{11} aromatics	5.0
		C_{12} aromatics	0.8
Carbon balance (%)	74.7	Carbon balance (%)	92.0

Supplementary Fig. 36 Sankey diagram based on carbon balance in the conversion of hemicellulose to different products. CPO: cyclopentanone. CPL: cyclopentanol. THFA: tetrahydrofurfuryl alcohol. MTHF: methyltetrahydrofuran. WHSV: weight hour space velocity. [a] Carbon yield calculated based on hemicellulose. [b] Carbon yield calculated based on CPO.

Supplementary Table 8. The practical processes for the synthesis of jet fuel range hydrocarbons with xylose or hemicellulose.

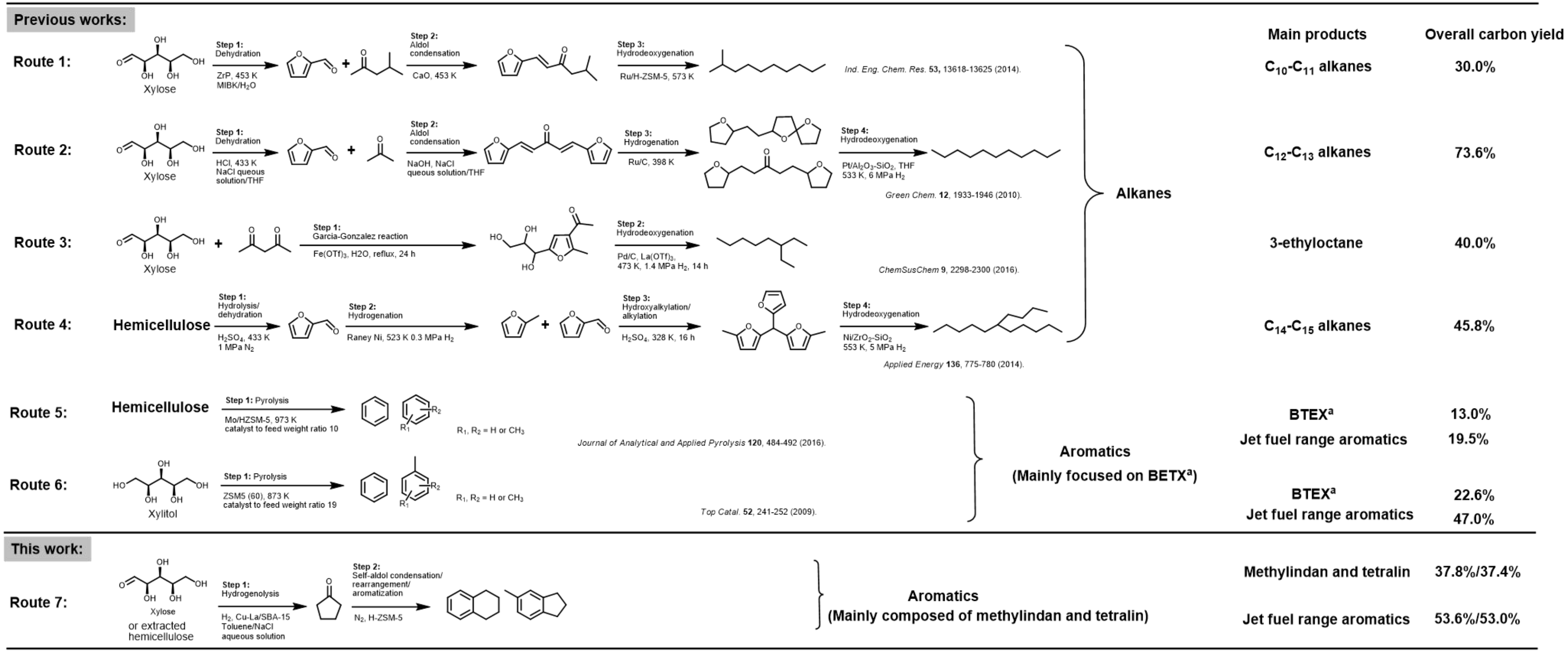

[a] BTEX: benzene, toluene, ethylbenzene and xylenes.

Supplementary Table 9 The overall carbon yields for the synthesis of jet fuel range hydrocarbons with xylose or hemicellulose.

	Substrates	Reaction steps	Products and carbon yield						Reference
			Products	Overall carbon yield (%)	Main products[c]	Carbon yield (%)	Other products	Carbon yield (%)	
Route 1	Xylose + MIBK[a]	3	C_8-C_{11} alkanes	30.5	C_{10}-C_{11} alkanes	~30.0	C_8-C_9 alkanes	~0.5	1
Route 2	Xylose + acetone	4	C_8-C_{13} alkanes	76.0	C_{12}-C_{13} alkanes	73.6	C_8-C_{11} alkanes	2.4	2
Route 3	Xylose + acetylacetone	2	C_{10} alkane and C_{12} oxygenate	68.0	3-ethyloctane	40.0	C_{12} oxygenate	28.0	3
Route 4	Hemicellulose	4	C_8-C_{15} alkanes	49.8	C_{14}-C_{15} alkanes	45.8	C_8-C_{13} alkanes	4.0	4
Route 5	Hemicellulose	1	Aromatics	19.5	BTEX	13.0	Benzene derivatives<br>Polyaromatics	2.3<br>4.2	5
Route 6	Xylitol	1	Aromatics	47.0	BTEX	22.6	Substituted benzene<br>Indanes<br>Naphthalenes	2.1<br>2.6<br>18.8	6
Route 7	Xylose	2	Aromatics	53.6	Methylindan and tetralin	37.8	BTEX[c] and C_{11}-C_{12} aromatics	15.8	This work
	Hemicellulose[b]	2	Aromatics	53.0	Methylindan and tetralin	37.4	BTEX[c] and C_{11}-C_{12} aromatics	15.6	

[a] MIBK: methyl isobutyl ketone. [b] Hemicellulose account for the hemicellulose solutions that were extracted from poplar wood. [c] BTEX: benzene, toluene, ethylbenzene and xylenes.

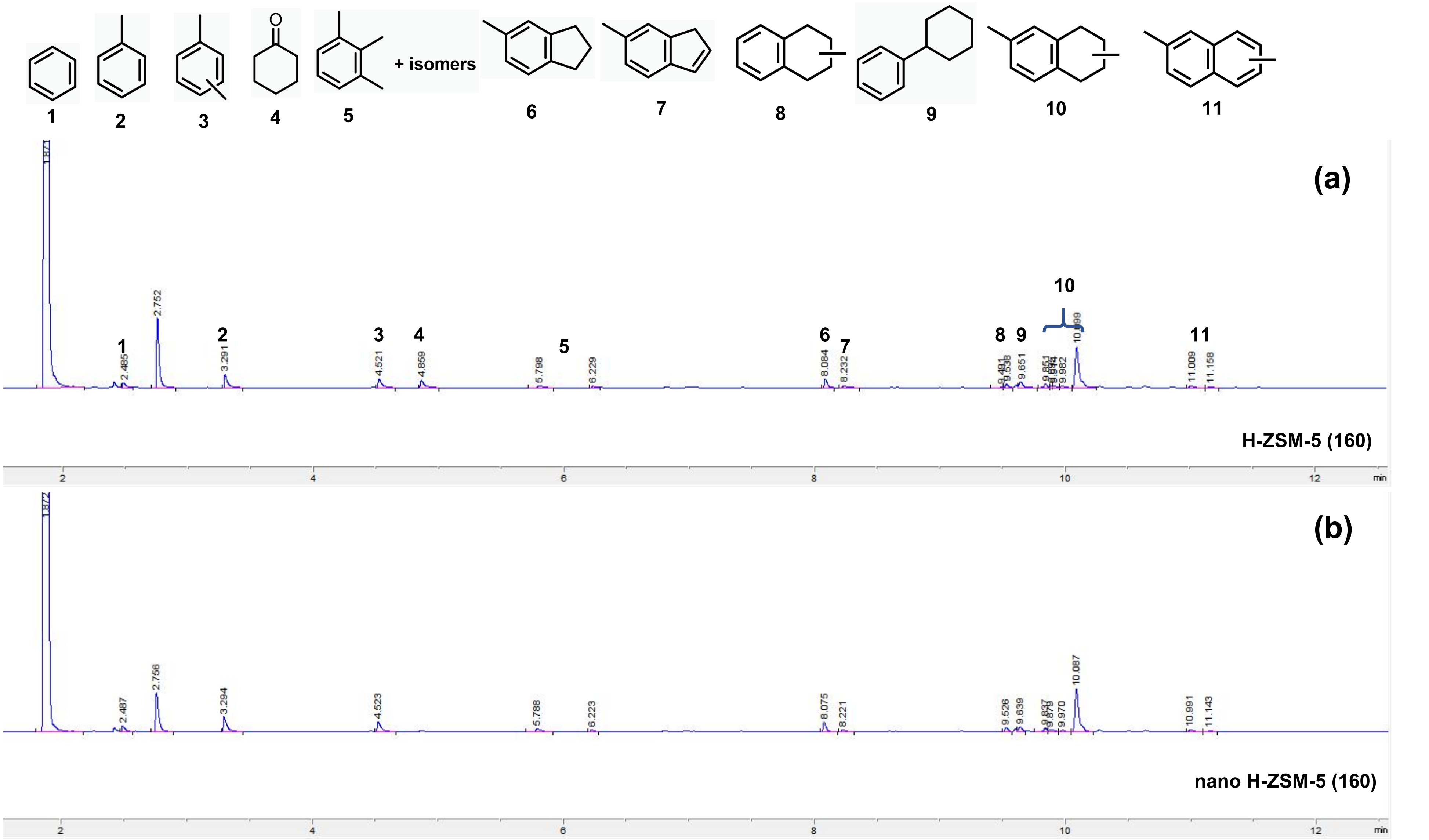

Supplementary Fig. 37 GC chromatograms of the products that were obtained from the reaction of CHO over the (a) H-ZSM-5 (160) and (b) nano H-ZSM-5 (160) catalysts. Reaction conditions: 0.1 MPa N_2, 663 K, WHSV = 0.6 g g^{-1} h^{-1}, the initial N_2/CHO molar ratio = 36/1. CHO: cyclohexanone. WHSV: weight hour space velocity.

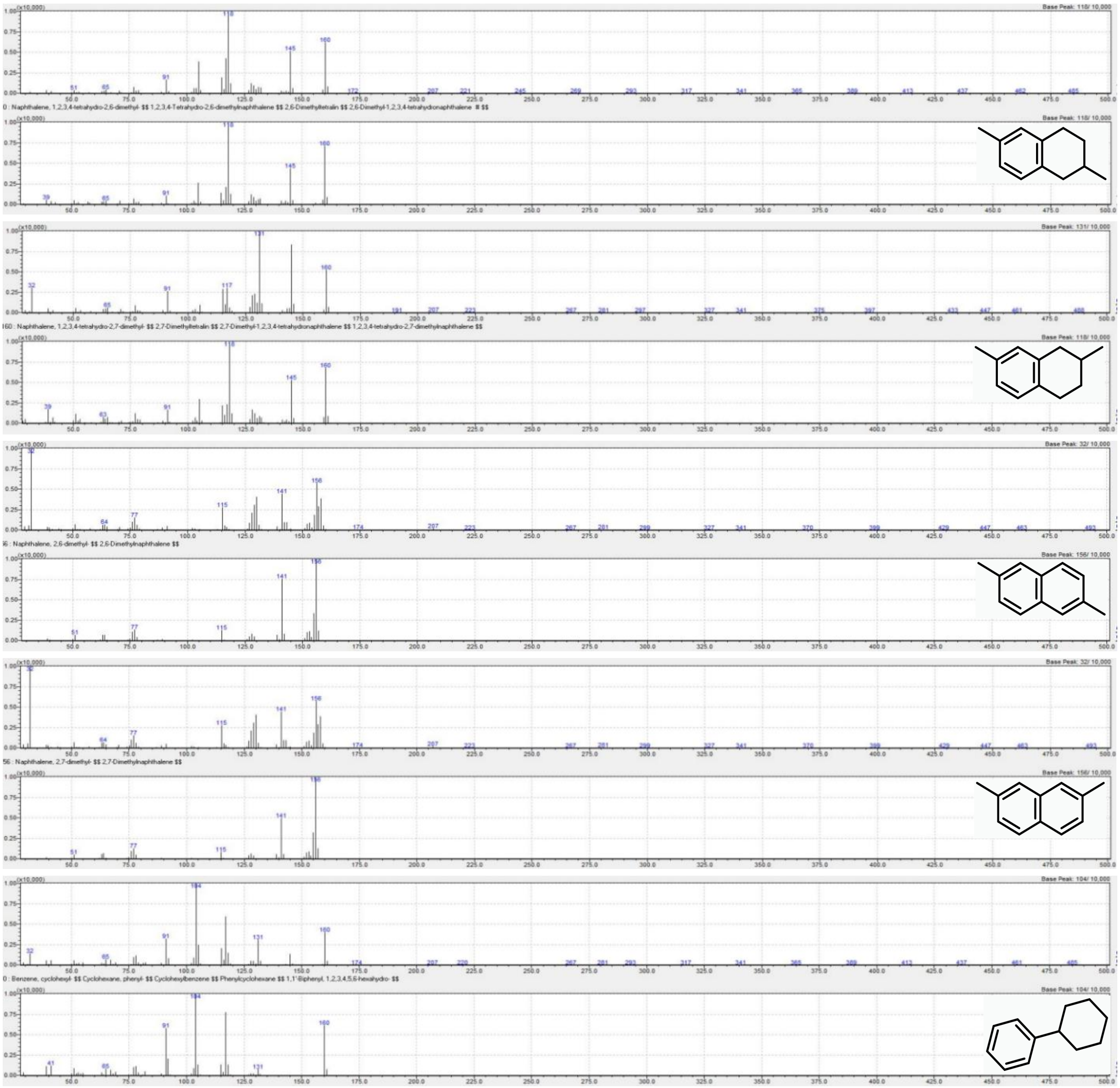

Supplementary Fig. 38 Mass spectrograms of the C_{12} aromatics obtained from the reaction of CHO over the H-ZSM-5 (160) and nano H-ZSM-5 (160) catalysts. Reaction conditions: 0.1 MPa N_2, 663 K, WHSV = 0.6 g g^{-1} h^{-1}, the initial N_2/CHO molar ratio = 36/1. CHO: cyclohexanone. WHSV: weight hour space velocity.

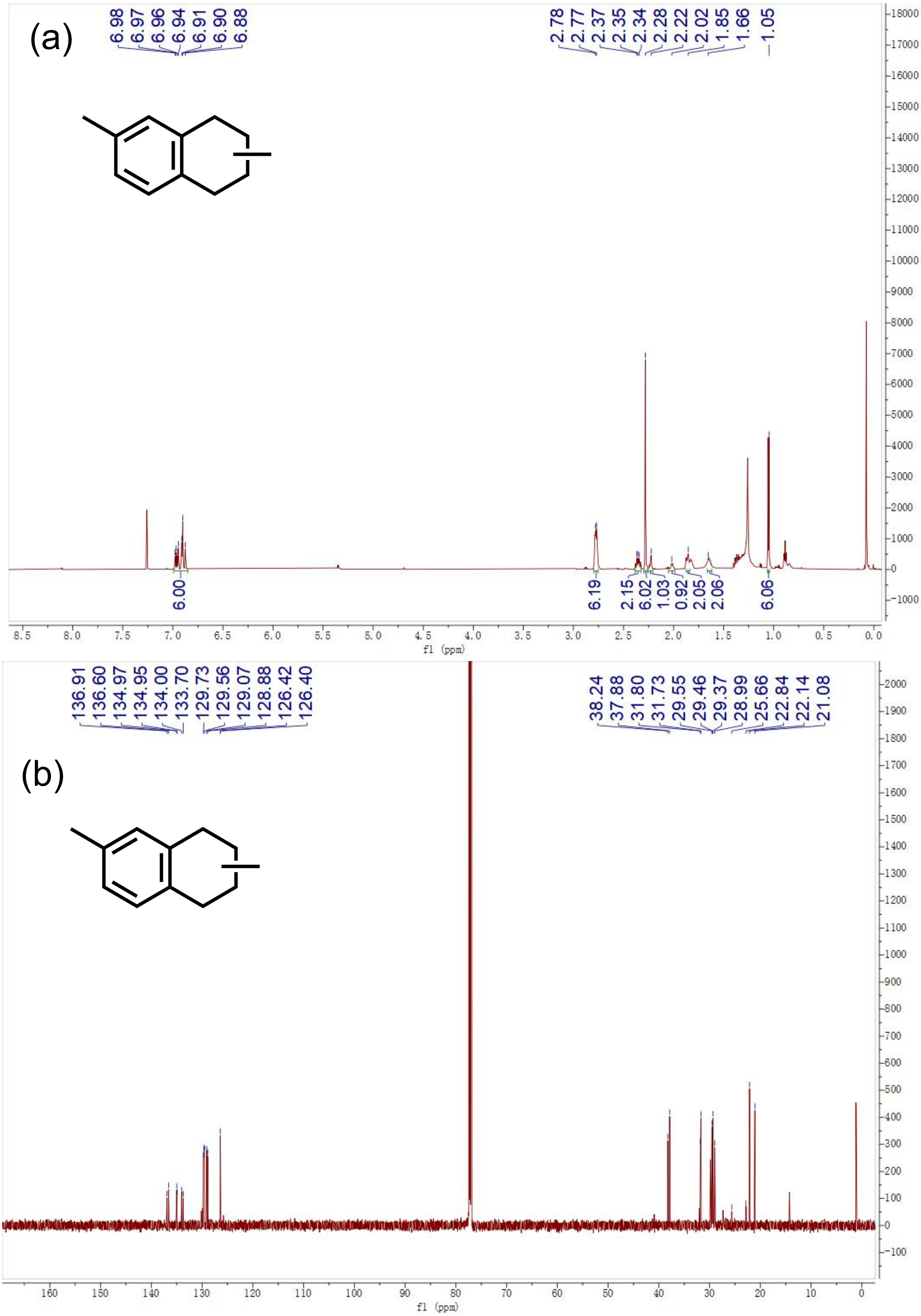

Supplementary Fig. 39 ^{1}H NMR (a) and ^{13}C NMR (b) spectra of dimethyltetralin obtained from the reaction of CHO over the H-ZSM-5 (160) catalyst. CHO: cyclohexanone.

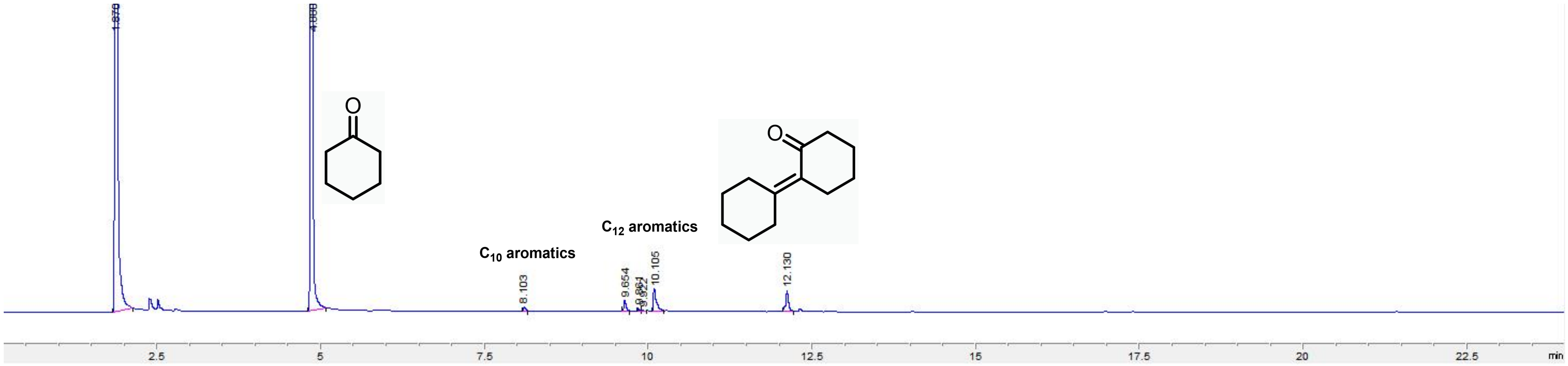

Supplementary Fig. 40 GC chromatogram of the products obtained from the reaction of CHO over the H-ZSM-5 (160) catalyst at a relatively low reaction temperature (613 K) and and a relatively high WHSV (1.2 g g^{-1} h^{-1}). Reaction conditions: 0.1 MPa N_2, 613 K, WHSV = 1.2 g g^{-1} h^{-1}, the initial N_2/CHO molar ratio = 36/1. CHO: cyclohexanone. WHSV: weight hour space velocity.

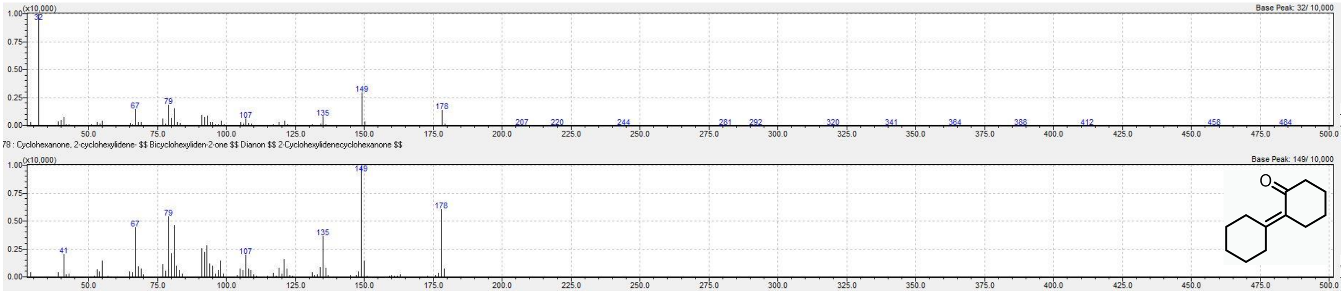

Supplementary Fig. 41 Mass spectrogram of the [1,1'-bi(cyclohexylidene)]-2-one obtained from the reaction of CHO over the H-ZSM-5 (160) catalyst at a relatively lower reaction temperature and a relatively higher WHSV. Reaction conditions: 0.1 MPa N_2, 613 K, WHSV = 1.2 g g^{-1} h^{-1}, the initial N_2/CHO molar ratio = 36/1. CHO: cyclohexanone. WHSV: weight hour space velocity.

Supplementary References

1. Pholjaroen, B. et al. Production of renewable jet fuel range branched alkanes with xylose and methyl isobutyl ketone. *Ind. Eng. Chem. Res*. **53**, 13618-13625 (2014).
2. Xing, R. et al. Production of jet and diesel fuel range alkanes from waste hemicellulose-derived aqueous solutions. *Green Chem*. **12**, 1933-1946 (2010).
3. Sutton, A. D. et al. The conversion of starch and sugars into branched C_{10} and C_{11} hydrocarbons. *ChemSusChem* **9**, 2298-2300 (2016).
4. Wang, T. et al. Aviation fuel synthesis by catalytic conversion of biomass hydrolysate in aqueous phase. *Appl. Energy* **136**, 775-780 (2014).
5. Yang, Z. X., Kumar, A. & Apblett, A. Integration of biomass catalytic pyrolysis and methane aromatization over Mo/HZSM-5 catalysts. *J. Anal. Appl. Pyrol.* **120**, 484-492 (2016).
6. Carlson, T. R., Tompsett, G. A., Conner, W. C. & Huber, G. W. Aromatic production from catalytic fast pyrolysis of biomass-derived feedstocks. *Top Catal.* **52**, 241-252 (2009).
